# Activation of the Dormant Secondary Metabolite Production by Introducing Gentamicin-Resistance in a Marine-Derived *Penicillium purpurogenum* G59

**DOI:** 10.3390/md10030559

**Published:** 2012-03-02

**Authors:** Yun-Jing Chai, Cheng-Bin Cui, Chang-Wei Li, Chang-Jing Wu, Cong-Kui Tian, Wei Hua

**Affiliations:** Beijing Institute of Pharmacology and Toxicology, 27 Tai-Ping Road, Haidian District, Beijing 100850, China; Email: chaiyunjing@hotmail.com (Y.-J.C.); sdrlcw@sohu.com (C.-W.L.); wucj2009@163.com (C.-J.W.); tiantian200102@163.com (C.-K.T.); huawei1980@yahoo.cn (W.H.)

**Keywords:** *Penicillium purpurogenum* G59, marine-derived fungus, gentamicin resistance, DMSO, antitumor activity, secondary metabolite production

## Abstract

A new approach to activate silent gene clusters for dormant secondary metabolite production has been developed by introducing gentamicin-resistance to an originally inactive, marine-derived fungal strain *Penicillium purpurogenum* G59. Upon treatment of the G59 spores with a high concentration of gentamicin in aqueous DMSO, a total of 181 mutants were obtained by single colony isolation. In contrast to the strain G59, the EtOAc extracts of nine mutant cultures showed inhibitory effects on K562 cells, indicating that the nine mutants had acquired capability to produce antitumor metabolites. This was evidenced by TLC and HPLC analysis of EtOAc extracts of G59 and the nine mutants. Further isolation and characterization demonstrated that four antitumor secondary metabolites, janthinone (**1**), fructigenine A (**2**), aspterric acid methyl ester (**3**) and citrinin (**4**), were newly produced by mutant 5-1-4 compared to the parent strain G59, and which were also not found in the secondary metabolites of other *Penicillium purpurogenum* strains. However, Compounds **1**–**4** inhibited the proliferation of K562 cells with inhibition rates of 34.6% (**1**), 60.8% (**2**), 31.7% (**3**) and 67.1% (**4**) at 100 μg/mL, respectively. The present study demonstrated the effectiveness of a simple, yet practical approach to activate the production of dormant fungal secondary metabolites by introducing acquired resistance to aminoglycoside antibiotics, which could be applied to the studies for eliciting dormant metabolic potential of fungi to obtain cryptic secondary metabolites.

## 1. Introduction

Natural products are important sources of new drugs and drug leads [1,2,3,4]. It has been reported that approximately 50% of new drugs approved from 1981 to 2006 are natural molecules or derived therefrom [4]. A significant number of natural product drugs and leads are of microbial origin [4,5,6,7], among which fungal secondary metabolites are of particular importance. Research on fungal secondary metabolites has attracted considerable attention [8,9,10,11,12,13,14,15] with particular interest in marine-derived fungi [10,11,12,13,14] and an increasing number of reports are related to the production of bioactive metabolites [13,14,15,16,17,18,19,20,21,22,23]. Secondary metabolites from uncultured microorganisms have now become accessible by the metagenomics method, bypassing the isolation and cultivation processes [24,25,26], or by new cultivation approaches [25,26]. In spite of this, cultured microorganisms are still a leading source of bioactive metabolites.

However, the majority of cultured microbes often do not produce bioactive metabolites under the laboratory culture conditions and thus could not be used for producing strains of anticipated metabolites. A number of these strains were thus stocked or even destroyed. Although these strains indeed possess potential for the production of bioactive metabolites, the gene clusters remain inactive under general culture conditions [26,27,28,29]. An increasing number of the predicted biosynthetic genes from genome sequencing of both bacteria and fungi [30,31,32,33,34,35,36] have demonstrated that the genome-based biosynthetic potential for secondary metabolite production in bacteria and fungi is a long way from being known for a given organism by chemical examination of the secondary metabolites. The impact of microbial genomics on natural product research has yet to be taken into serious consideration [37,38]. The regulation of fungal secondary metabolism has also been explored to a certain extent, from genetic, genomic and biochemical perspectives, in transcriptional, translational and enzymatic levels [16,17,18,19,20,21,22,23]. Various genetic strategies have recently been developed to activate the silent gene clusters in order to obtain cryptic secondary metabolites [26,27,28,29]. However, the requirement for complex gene manipulation in those approaches has restricted their applications in general microbial product research.

In contrast, the one-strain-many-compounds (OSMAC) strategy [39] has been widely applied in searching bioactive microbial metabolites as a simple and efficient approach to improve metabolic diversity [15,39,40,41]. The OSMAC approach elicits the metabolic potentials of microorganisms to produce diverse metabolites, including new compounds, by variation of the fermentation parameters or medium compositions. Similarly, the chemical epigenetics methodology has offered a new approach that enables chemists to simply access potential pools of cryptic fungal secondary metabolites [42,43,44,45,46]. The chemical epigenetics approach manipulates epigenetic processes by administering small-molecule epigenetic modifiers to fungal cultures to induce silent biosynthetic pathway transcription to activate the production of cryptic metabolites, including new compounds. Differing in the variation of culture conditions in the OSMAC approach or in adding small-molecule epigenetic modifiers to fungal cultures in the chemical epigenetics approach, ribosome engineering [47,48] has also provided a simple way to activate cryptic biosynthesis pathways of secondary metabolites by introducing drug-resistant mutation in bacteria [26,49]. However, application of this approach to fungi has not been reported.

We have thus undertaken a research work to develop a similar mutation-based new approach to activate silent gene clusters to awake dormant secondary metabolite production in fungi. Several wild-type fungal strains from different environments were selected for the aimed study, including a marine-derived *Penicillium purpurogenum* G59 [50] and a terrestrial *Penicillium aurantiogriseum* H4 [50]. These strains were isolated initially by our group from environmental samples and were originally inactive and thus unable to produce antitumor secondary metabolites in the MTT assay using K562 cells [50]. However, using these strains, we have established a new approach to activate dormant secondary metabolite production by introducing antibiotic-resistance to obtain antitumor secondary metabolites. Owing to its simplicity, this approach could also be applied in a general microbial laboratory to improve metabolic diversity for fungal metabolite studies.

Herein, we report in detail recent results of our research on *Penicillium purpurogenum* G59 using an aminoglycoside antibiotic, gentamicin.

## 2. Results

### 2.1. Finding Conditions for Introducing Gentamicin-Resistance in *Penicillium purpurogenum* G59

Gentamicin was used in ribosome engineering to introduce drug-resistance in bacteria. However, it could not be applied to fungi up to now because of its insensitivity. The fungus *Penicillium purpurogenum* G59 is also insensitive to gentamicin. We assumed that low intracellular concentration of gentamicin is likely to be a possible reason for its insensitiveness to fungi and furthermore the permeability of the fungal membrane to gentamicin may restrict its intracellular concentration. Dimethylsulfoxide (DMSO) is known as a membrane permeabilizer which enhances the penetration of both hydrophilic and hydrophobic molecules into the cells. Thus, DMSO was chosen as first choice accessorial agent to find conditions for introducing gentamicin-resistance into the fungal strain G59 in the present study.

**Table 1:** Group settings of G59 spore suspensions for Tests in 20% and 50% dimethylsulfoxide (DMSO).

Tests in	Test and Control Groups^ a^	Gentamicin (mg/mL)	Dilution Ratio ^b^
20% DMSO	Tests in 20% DMSO with gentamicin	0.5, 1, 2, 5, 10, 20, 50, 100	OSS:DMSO = 4:1
	Control in 20% DMSO without gentamicin	0	OSS:DMSO = 4:1
	Controls in water with gentamicin	0.5, 1, 2, 5, 10, 20, 50, 100	OSS:H_2_O = 4:1
	Control in water without gentamicin	0	OSS:H_2_O = 4:1
50% DMSO	Tests in 50% DMSO with gentamicin	1, 2, 5, 10, 20	OSS:DMSO = 1:1
	Control in 50% DMSO without gentamicin	0	OSS:DMSO = 1:1
	Controls in water with gentamicin	1, 2, 5, 10, 20	OSS:H_2_O = 1:1
	Control in water without gentamicin	0	OSS:H_2_O = 1:1

^a^ Spore suspensions in the test and control groups were prepared using the same original G59 spore suspension by dilution with DMSO or water in the same ratio for the 20% and 50% DMSO groups, respectively, so that the spore density remained the same in the 20% and 50% DMSO groups, respectively. Details see in Experimental Section; ^b^ OSS in this column indicates the original G59 spore suspension in water.

Search of experimental condition for introducing gentamicin-resistance, as well as study of the effects of gentamicin, DMSO and their combinations on the growth of G59 were undertaken at first, after treatment of the G59 spores in the test and control groups in 20% and 50% DMSO (Table 1) at 4 °C for 1–20 days. The treated spores were spread on potato dextrose agar (PDA) plates and incubated at 28 °C to estimate the growth of G59.

**Figure 1:**
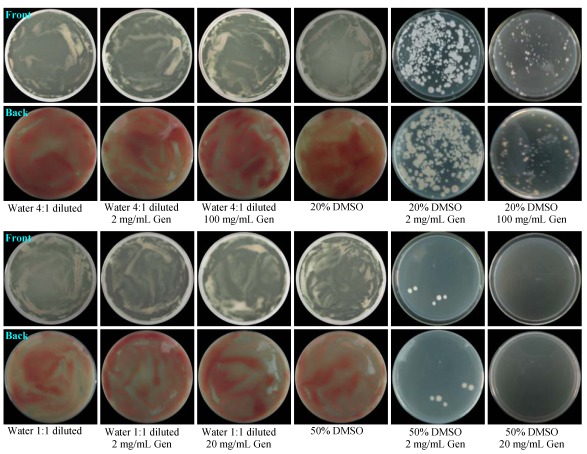
The growth of *Penicillium purpurogenum* G59 on PDA plates by incubation at 28 °C for 3 days after pretreatment of the G59 spores at 4 °C for 3 days. Left four columns show controls and right two columns show test groups. Descriptions under the photographs indicate the spore pretreatment conditions. Gen is the abbreviation of gentamicin.

The pretreatment of G59 spores at 4 °C in water with gentamicin or in 20% and 50% DMSO without gentamicin did not affect the growth of G59 on PDA plates at 28 °C. Typical examples as shown in Figure 1, of G59 in the control groups grew well throughout on the plate. However, pretreatment of the spores with gentamicin at 4 °C in 20% and 50% DMSO resulted in significant inhibition of G59 growth at 28 °C on PDA plates, allowing the development of resistant mutant colonies. Thus, the conditions and experimental procedure for introducing gentamicin-resistance were decided as follows: First, the G59 spores were treated with the mg/mL amount of gentamicin in 20% or 50% DMSO (Table 1) at 4 °C for 1–20 days. Second, the resistant mutants were selected by single colony isolation using the pretreated G59 spores.

**Figure 2:**
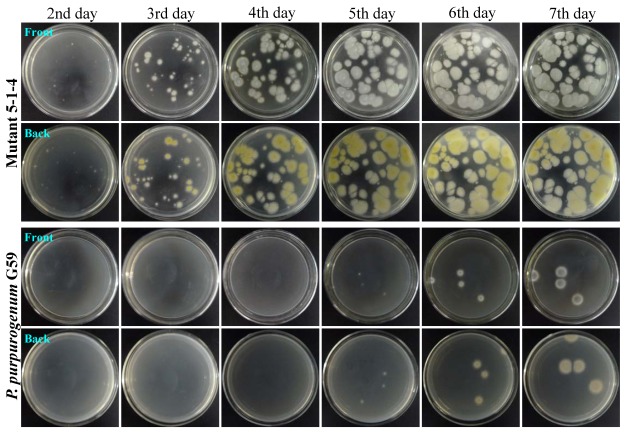
The growth of *Penicillium purpurogenum* G59 and its mutant 5-1-4 on PDA plates by incubation at 28 °C for different times (day) after treatment of their spores with gentamicin. The G59 and 5-1-4 spore suspensions with 5 mg/mL gentamicin in 50% DMSO at the same spore density were stored at the same 4 °C condition for 1 day to treat the spores with gentamicin. Then, each 100 μL of the treated spore suspensions was spread on PDA plates, incubated at 28 °C, and photographed at the given incubation times (day).

The resistance of the mutants to gentamicin was testified by a resistance test using a mutant 5-1-4 and the parent G59. After pretreatment of the 5-1-4 and G59 spores with 5 mg/mL gentamicin as that for selecting the mutant 5-1-4, both the spores were spread on PDA plates and incubated at 28 °C for 10 days to examine their day-by-day growth. The colonies of mutant 5-1-4 appeared on the second day of incubation and developed to become confluent with the incubation times as shown in Figure 2. In contrast, few colonies of G59 appeared on the fifth day of incubation and developed to give four isolated colonies on the sixth day of incubation (Figure 2), which were kept isolated for the incubation time period examined. These results demonstrated that the treatment of G59 spores with gentamicin in the present study successfully introduced the gentamicin-resistance into the mutant 5-1-4.

### 2.2. Mutant Selection

As shown in Figure 1, the pretreatment of G59 spores with gentamicin in aqueous DMSO resulted in the development of resistant colonies on PDA plates. Thus, single colony isolation was performed using G59 spores treated with gentamicin in 20% and 50% DMSO at 4 °C for different times by spreading them on PDA plates. The colonies selected are gentamicin-resistant mutants, having survived by acquiring resistance capability to the treatment. A total of 181 mutants were selected with different appearances of colonies on PDA plates, including 136 and 45 mutants from 20% and 50% DMSO test groups, respectively (Table 2).

**Table 2:** Mutant selection by gentamicin-treatment of G59 spores at 4 °C for different times in the presence of DMSO ^a^.

DMSO% (v/v)	Gentamicin (mg/mL)	Numbers of Mutant Strains Selected on the Days of Gentamicin-Treatment at 4 °C						
		1st d	3rd d	5th d	10th d	15th d	20th d	Total
20%	0.5	1	1	3	NS	2	ND	7
	1	1	1	NS	1	6	ND	9
	2	1	1	NS	4	12	ND	18
	5	1	1	3	3	2	ND	10
	10	1	2	10	NS	7	ND	20
	20	1	1	3	27	1	ND	33
	50	1	1	10	4	7	ND	23
	100	1	1	4	5	5	ND	16
	Sum	8	9	33	44	42	0	136
50%	1	NS	NC	NC	1	NC	NC	1
	2	9	4	5	8	NC	8	34
	5	4	NC	NC	NC	NC	NC	4
	10	NS	NC	NC	2	NC	NC	2
	20	4	NC	NC	NC	NC	NC	4
	Sum	17	4	5	11	0	8	45
Total		25	13	38	55	42	8	181

^a^ Spores of the strain G59 in 20% and 50% DMSO aqueous suspensions were treated with gentamicin at the given concentration at 4 °C for different times. During the treatment period, each 90 μL of the spore suspensions treated was spread on PDA plates, incubated at 28 °C for 3–4 days, and single colonies formed were selected with different appearances of colonies to obtain the mutant strains; NS: No single colonies could be selected due to their growth throughout on the plate as the growth for controls. ND: Not done; NC: No colonies developed.

### 2.3. Phenotypes of G59 Mutants

**Figure 3:**
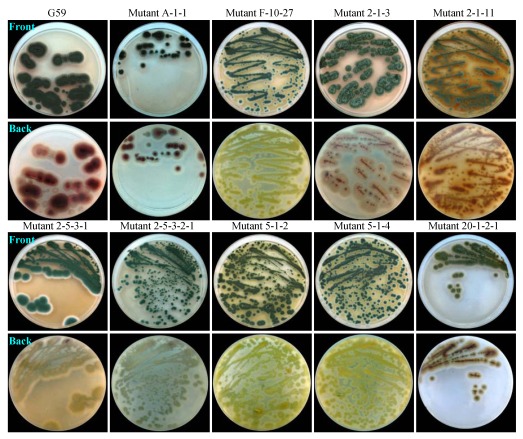
Phenotypes of the parent strain G59 and its mutants growing on PDA plates by incubation at 28 °C for 3 days.

Both growth and spore formation of 181 of the mutants were not so obviously different from those of the parent strain G59 under the culture conditions at 28 °C on PDA plates. However, they showed obviously different morphology, color of mycelia, or pigment formation, when grown on PDA plates at 28 °C (typical examples are shown in Figure 3). These differences correlated with the different appearances of corresponding colonies observed during mutant selection, which were used as an indicator to select different mutants in single colony isolation. Correspondingly, similar differences in colors of mycelia and culture broths were also observed by fermentation in liquid medium (data not shown).

### 2.4. Inhibitory Effect of G59 Mutant Samples on K562 Cells

All EtOAc extracts from the fermentation of G59 and its mutants were subjected to MTT assay at 100 μg/mL. In triplicate MTT tests using samples from three rounds of individual fermentations, nine mutants showed inhibitory effects on the proliferation of K562 cells with mean IR% values over 40% (Table 3). Among them, two were from the 20% DMSO group, which accounts for 1.5% of the 136 mutants from this group; whereas seven were from the 50% DMSO group, accounting for 15.6% of the 45 mutants from this group.

**Figure 4:**
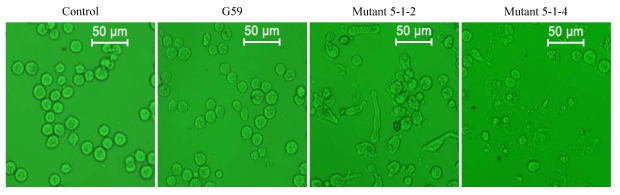
Morphological observations of the K562 cells treated with 100 μg/mL samples at 37 °C for 24 h (×200).

Prior to MTT treatment of K562 cells in the MTT assay, the morphology of K562 cells treated with 100 μg/mL of EtOAc extracts was observed under a reversed phase microscope. Corresponding to the MTT assay results (Table 3), morphological observations (full data not shown) revealed that the K562 cells treated with a G59 sample showed no morphological changes in comparison with the control (Figure 4). However, a portion of the cells treated with a mutant 5-1-2 sample showed heteromorphosis with club-shaped cells, revealing an effect of the sample on cytoskeleton of inhibiting cell division (Figure 4). Furthermore, a major portion of the cells treated with the other eight mutant samples showed necrotic cell morphology, as shown for mutant 5-1-4 in Figure 4, indicating cytotoxic effects of the eight mutant samples on K562 cells.

**Table 3:** MTT assay results on K562 cells for G59 and its mutant samples at 100 μg/mL ^a^.

No.	Strain	Condition for Selecting Mutant Strain			IR% (Mean ± SD, *n *= 3)^ b^
		DMSO (%)	Gentamicin (mg/mL)	Treated Time (days)	
0	G59	—	—	—	5.8 ± 0.5
1	A-1-1	20%	0.5	1	42.0 ± 12.7
2	F-10-27	20%	20	10	45.5 ± 10.8
3	2-1-3	50%	2	1	43.5 ± 10.3
4	2-1-11	50%	2	1	40.0 ± 7.3
5	2-5-3-1	50%	2	5	80.7 ± 0.7
6	2-5-3-2-1	50%	2	5	46.1 ± 16.1
7	5-1-2	50%	5	1	47.2 ± 19.8
8	5-1-4	50%	5	1	43.7 ± 2.8
9	20-1-2-1	50%	20	1	52.2 ± 18.1

^a^ Test samples are the EtOAc extracts of G59 and its mutant cultures; ^b^ Triplicate MTT tests were carried out using samples from three rounds of individual fermentations, respectively. Among the 181 mutant samples tested, nine given in this table showed an inhibitory effect on K562 cells in contrast to G59, whereas the other ones not listed in this table showed no effect on K562 cells. IR% values for several mutants showed quite a variation with the samples from individual fermentations.

### 2.5. TLC and HPLC Analysis of the EtOAc Extracts of G59 and Its Nine Mutant Cultures

**Figure 5:**
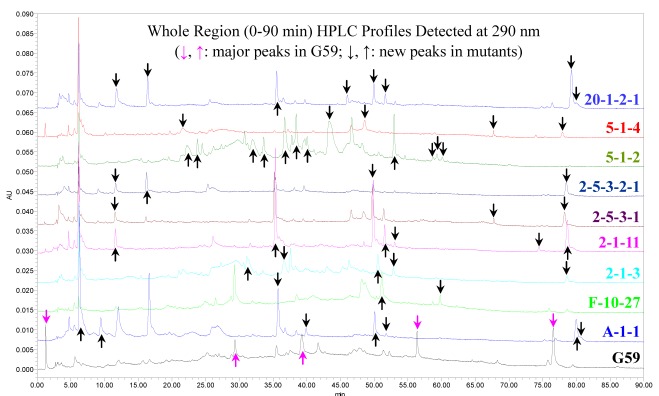
HPLC metabolite profile comparison in G59 and selected mutant strains. The EtOAc extracts used in the HPLC analysis were from the third fermentation at the same time and same conditions, which were used also for the third test of MTT assay.

The EtOAc extracts from G59 and nine mutants in Table 3, prepared for the third test of MTT assay, were subjected to TLC and HPLC analysis. By comparison with the strain G59, several major components detected in the G59 sample by HPLC disappeared in all nine mutant samples almost completely, and instead, the secondary metabolites newly produced by the mutants were detected both by TLC (Supplementary Data S1) and HPLC (Figure 5 and Supplementary Data S1). HPLC profiles detected at different wave lengths and TLC chromatograms in Supplementary Data S1 provided more detailed information including differences between one another. These results revealed that the introduction of gentamicin-resistance resulted in the alteration of secondary metabolisms to activate the production of some dormant secondary metabolites in G59.

### 2.6. Producing Fermentation, Isolation and Identification of Antitumor Metabolites ***1–4***

During the course of mutant 5-1-4 fermentation, relative percentage of the mycelial volume and inhibitory effect of the EtOAc extract at 100 μg/mL reached their maxima at 11 and 12 days of the fermentation, respectively, while pH of the supernatants (pH 6.0) remained unchanged throughout fermentation period tested. Thus, the producing fermentation of mutant 5-1-4 was carried out for 12 days to obtain total 49 L of liquid cultures for the isolation of antitumor metabolites.

**Figure 6:**
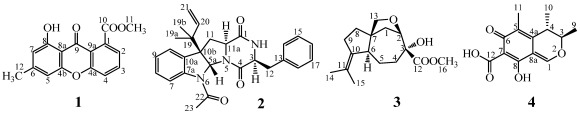
Chemical structures of **1**–**4** from the G59 mutant 5-1-4.

Total liquid cultures of mutant 5-1-4 were extracted with EtOAc to obtain an active EtOAc extract. The EtOAc extract was then separated by chromatographic means, tracing active components absent in the metabolites of G59 by direct comparison with the sample from G59, under guidance of bioassay and chemical examination. This has resulted in the isolation of four antitumor metabolites **1**–**4** (Figure 6) newly produced by the mutant 5-1-4 (see Supplementary Data S2).

Compound **1** giving *pseudo*-molecular ion peak at *m/z* 285 [M + H]^+^ in ESI-MS and compound **2**, [α]^20^_D_ −175.4 (*c* 0.24, CHCl_3_), showing *pseudo*-molecular ion peak at *m/z* 444 [M + H]^+^ in ESI-MS were identified as janthinone [51] (**1**, Figure 6) and fructigenine A [52] (**2**, Figure 6) on the basis of their NMR data, respectively.

Compound **3** was obtained as a colorless oil with [α]^20^_D_ −59 (*c* 1.0, MeOH) and its molecular formula could be determined as C_16_H_24_O_4_ by HR-ESI-MS measurement. The ^1^H and ^13^C NMR spectra of **3** in CD_3_OD closely resembled those of aspterric acid [53], except for additional signals ascribable to a methoxy group (δ_H_3.72 and δ_C_52.4), and detailed analysis of its DEPT, ^1^H–^1^H COSY, HMQC and HMBC spectra led to the assignment of **3** as aspterric acid methyl ester.

Compound **4**, [α]^20^_D_ −17.3 (*c* 1.0, EtOH), giving *pseudo*-molecular ion peak at *m/z* 251 [M + H]^+^ in ESI-MS, could be identified as citrinin [54,55] based on its ^1^H and ^13^C NMR data.

### 2.7. Inhibitory Effect of Compounds ***1–4*** on the Proliferation of K562 Cells

The antitumor activity of **1**–**4** was tested preliminarily by the MTT assay using K562 cells, coupled with the morphological examination of K562 cells under a reversed phase microscope. In MTT assay, all of **1**–**4** showed to a certain extent an inhibitory effect on K562 cells in the inhibition intensity order of **4** > **2** > **1** and **3** (Table 4), and the IC_50_ values for **4** and **2** could be determined as 52.6 and 58.4 μg/mL, respectively. Morphological examination of the K562 cells treated with **1**–**4** at 100 μg/mL showed that **1**–**4** could inhibit K562 cells mainly by their cytotoxic effect except for an additional, weak but significant effect of **4** on cytoskeleton to inhibit cell division, revealed by a small portion of club-shaped K562 cells similar to those shown for mutant 5-1-2 in Figure 4.

**Table 4:** Inhibitory effect of **1**–**4** on the proliferation of K562 cells assayed by MTT method.

Compound	IR% at the Given Concentration (μg/mL)						IC_50_ (μg/mL)
	100 μg/mL	50 μg/mL	25 μg/mL	12.5 μg/mL	6.25 μg/mL	3.125 μg/mL	
**1**	34.6	28.4	20.6	14.3	12.6	7.6	>100
**2**	60.8	43.1	37.7	35.9	31.6	22.7	58.4
**3**	31.7	20.8	17.5	14.1	8.9	6.7	>100
**4**	67.1	44.6	41.6	25.0	2.3	1.1	52.6

## 3. Discussions

Gentamicin is one of the aminoglycoside antibiotics that target microbial ribosome thereby interfering with protein synthesis. Aminoglycosides and other antibiotics that attack the ribosome have been used in bacterial ribosome engineering to obtain drug-resistant mutants overproducing antibiotics. It has been shown that the cellular function of those mutants has been altered by modulating their ribosomal functions at translational or transcriptional level [47,48]. The production of known [56,57,58,59,60] or new [49] dormant metabolites by the drug-resistant mutants has been also demonstrated, which has provided a simple experimental approach to activate silent genes in bacteria. However, similar studies on fungi have not been reported. Indeed, aminoglycosides have not been used in ribosome engineering for fungi yet, probably because they are generally antibacterial [61]. The present work was thus undertaken with particular attention focused on development of a new approach to introduce the acquired resistance to aminoglycosides in fungi.

Aminoglycosides bind to the ribosomal RNA at the A site of the decoding region, causing codon misreading and translocation inhibition in both prokaryotic and eukaryotic ribosomes [62,63]. The structural differences in prokaryotic and eukaryotic rRNA cause the binding of aminoglycosides, more specifically, with at least a 10-fold higher affinity to prokaryotic rRNA than eukaryotic rRNA [64]. However, this difference has been addressed as not being so large, which may on one hand provide an explanation in part of the toxic effects of aminoglycosides on mammalian systems [61]. On the other hand, bacterial resistance to aminoglycosides arises broadly from: (a) deactivation of aminoglycosides by chemical modification; (b) reduction of intracellular antibiotic concentration by changing cellular functions associated with the antibiotic transfer process; (c) alteration of the 30S ribosomal subunit target by mutation; and (d) the 16S rRNA methylation at the aminoglycoside binding site [61]. Mutations on the 30S ribosomal subunit targets in bacteria has been reported also by ribosome engineering and certain mutations conferring antibiotic resistance, such as *rpsL* or *proB* mutations, have been found to be associated to a certain extent with the change of cellular function for secondary metabolite production [47,48,49,56,57,58,59,60]. Some aminoglycosides have also been reported to show antifungal activity against several plant pathogens within species or genera of oomycetes, but not against *Penicillium* and *Aspergillus* species tested [65]. Indeed, gentamicin did not show any inhibitory effect on *P. purpurogenum* G59 in our routine test using both aqueous solid and liquid media. Thus it is impossible to select drug-resistant mutants from strain G59 using gentamicin-containing plates based on the general method used to obtain drug-resistant mutants in bacterial ribosome engineering [47].

In view of the lower binding affinity of aminoglycosides to eukaryotic rRNA, it is reasonable to consider that the insensitivity of the fungal strain G59 to gentamicin may presumably result from low intracellular concentration of gentamicin. We thus attempted, at the early test stage of our study, to treat G59 with a high concentration of gentamicin in combination with chemical or physical factors that may affect the permeability of cellular membranes, such as alcohol, DMSO, acetone, microwave or ultrasonic radiation, etc., to obtain gentamicin-resistant mutants. We eventually found that treatment of G59 spores by combination of high concentrations of gentamicin and DMSO could inhibit growth of G59, allowing development of resistant colonies on PDA plates. This has enabled us to perform single colony isolation to a select 181 gentamicin-resistant mutants. The acquired resistance of the mutants to gentamicin was testified by the resistance test with a mutant 5-1-4 and the parent strain G59. After pretreatment of their spores with 5 mg/mL gentamicin as that for selecting mutant 5-1-4, the mutant 5-1-4 grew well on a PDA plate at 28 °C to form confluent lawn (Figure 2), whereas the growth of G59 was significantly inhibited so as to develop only four isolated colonies (Figure 2) as observed in the preliminary examination (Figure 1) and mutant selection. Thus the resistance test demonstrated that the gentamicin-resistance had been successfully introduced into the mutant 5-1-4.

**Figure 7:**
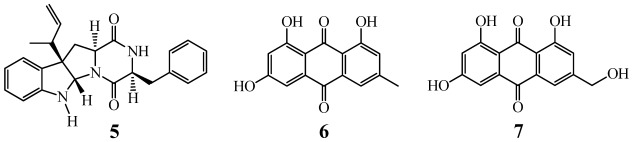
Chemical structures of **5**–**7** from the G59 mutant 2-5-3-1 [70].

The effect of introduction of gentamicin-resistance on the secondary metabolite production in G59 was demonstrated by both bioassay and chemical analysis. The EtOAc extract of the G59 culture did not show any inhibitory effect on K562 cells at 1000 µg/mL [50], whereas those from nine mutants inhibited significantly the proliferation of K562 cells at 100 µg/mL (Table 3), indicating that the secondary metabolism in the nine mutants has been altered to produce antitumor metabolites. TLC and HPLC analysis of the EtOAc extracts of G59 and nine mutant cultures also supported the altered secondary metabolite production in these mutants (Supplementary Data S1). This was further demonstrated by followed the elucidation of four antitumor secondary metabolites **1**–**4** (Figure 6) from one of the nine mutants, 5-1-4, in the present study. Compounds **1**–**4**, newly produced by mutant 5-1-4 compared to strain G59 (Supplementary Data S2), have also not been found yet in the secondary metabolites of other *P. purpurogenum* strains, and all they inhibited K562 cells to a different extent as shown in Table 4. A literature survey showed that no research report has been made on the antitumor activity of janthinone (**1**) and aspterric acid methyl ester (**3**). For fructigenine A (**2**), except for the report on its anti-inflammatory activity in mice and rats [66], the cytotoxic effect of **2** on murine L-5178Y and tsFT210 cells was also recorded [52,67], which coincided with its effect on human K562 cells in the present study. Citrinin (**4**) could induce apoptosis in both human HL-60 and Hep G2 cells [68,69], and its inhibitory effect on K562 cells [55] was reconfirmed by the present results (Table 4). Further support for the altered secondary metabolism in the nine mutants was also provided by previous work on another G59 mutant 2-5-3-1 (Table 3) used to elucidate antitumor secondary metabolites **5**–**7** (Figure 7) [70], newly produced by the mutant compared to the parent strain G59 (Supplementary Data S2). Compounds **5**–**7** were also not found in the secondary metabolites of other *P. purpurogenum* strains [70].

DMSO is an aprotic solvent with both hydrophilic and hydrophobic characteristics. It is not only soluble in both polar and nonpolar solvents, but also can solubilize a wide range of compounds. However, the solubility of gentamicin in aqueous DMSO decreased with an increase of DMSO, restricting the application of gentamicin to concentrations lower than 20 mg/mL in 50% DMSO spore suspension in the present study. Mild antibacterial and antifungal effects of DMSO at 37.5 °C have been reported [71]. DMSO is cellular protective at low temperature as a cryoprotectant, while at higher temperature it becomes toxic. The cellular toxicity of DMSO has been ascribed probably to its increasingly hydrophobic character at higher temperature. This makes the temperature-dependent hydrophobic interaction of DMSO with the hydrophobic region of the protein or membrane bilayer more favorable at higher temperature, resulting in destabilization of protein and cellular membrane at high temperature to show toxicity [72]. Coincidentally, DMSO induced a significant variation of metabolite production on adding 1–3% to growing bacterial cultures. However, the toxic effect of 5% or more DMSO to the producing strains cultured at 30 °C has been also reported [73]. Treatment of strain G59 at low temperature in the present study is thought likely to be a key point in overcoming the cellular toxicity of DMSO. G59 spores were treated at 4 °C by both 20% and 50% DMSO with or without gentamicin, and DMSO alone showed no effect on the growth of G59 on a PDA plate, while the combination of gentamicin with DMSO significantly inhibited the growth of G59, thereby affording 181 mutants. Therefore, the mutant selection does not rely on the inhibitory effect of DMSO itself [65], and the altered secondary metabolite production in the nine mutants is also not caused by the inducing effect of DMSO [73]. It should be noted that high concentrations of gentamicin in the mg/mL order were used in the present study to treat the G59 spores in combination with DMSO to select the mutants. In view of no direct effect of DMSO on G59 growth, high concentrations of gentamicin should play a major role in inhibition of the G59 growth, while DMSO is likely to exert its accessorial effect by modulating permeability of the cellular membrane [74]. Gentamicin is a strongly hydrophilic molecule, and its penetration into the cell membrane may be effectively enhanced by the enhancement mechanism of hydrophilic penetrant via formation of DMSO-induced water pores in the membrane bilayers, which requires high concentrations of DMSO [75], as also justified in the present study.

As mentioned, compounds **1**–**7** are newly produced by mutants 5-1-4 and 2-5-3-1 compared to their parent strain *P. purpurogenum* G59 (Supplementary Data S2), which have also not yet been found in the secondary metabolites of other *P. purpurogenum* strains. Compounds **1**–**7** belong to five different structural classes, indicating that at least five subsets of biosynthetic pathways for the **1**–**7** production have been activated in these mutants although detailed biological mechanisms remain unknown. Nevertheless, the above results have demonstrated that introduction of gentamicin-resistance by a high concentration of gentamicin in aqueous DMSO effectively activates dormant secondary metabolite production in strain G59, which could be useful in fungal metabolite studies as a simple, yet practical approach to elicit the biosynthetic potential of fungi in order to increase chemical diversity for drug screening. Activation mechanisms of dormant secondary metabolite production in the fungal mutants remain to be investigated: This includes whether or not *rpsL* mutations are concerned or involved in the activation processes, as certain *rpsL* mutations that confer resistance to aminoglycosides in bacteria have been known to activate secondary metabolite production [47,48,49,56], which has become of interest in our research subject.

According to the results that we have obtained and ongoing development of our research on G59 and other fungal strains with more aminoglycoside antibiotics (data will be reported elsewhere), application of the new approach reported in this paper could be expanded to more fungal strains, including those from deep sea environment with broad aminoglycoside antibiotics. This approach could also be applied by combination with other bioassays to improve more efficiently metabolic diversity in bioactive fungal metabolite studies.

## 4. Experimental Section

### 4.1. The Original Strain and Human Tumor Cell Line

The original strain *Penicillium purpurogenum* G59 used in the present study is a marine-derived wild-type fungal strain G59 isolated from a soil sample collected at the tideland of Bohai Bay around Lüjühe in Tianjin, China, in September, 2004 [50], which was identified later by L. Guo, Institute of Microbiology, Chinese Academy of Sciences, Beijing, China. The strain was deposited at the China General Microbiological Culture Collection Center under the accession number CGMCC No. 3560. The EtOAc extract of G59 fermentation culture did not show any effect on K562 cells and its inhibition rate at 1000 or 100 µg/mL was always lower than 6.9% in repeated tests using different samples from individually fermented cultures.

Human chronic myelogenous leukemia cell line K562 was provided by S. Li (Beijing Institute of Pharmacology and Toxicology). The cells were routinely maintained at 37 °C in RPMI-1640 medium supplemented with 10% (v/v) fetal bovine serum in the presence of 100 µg*/*mL penicillin and streptomycin under a humidified atmosphere of 5% CO_2_ and 95% air.

### 4.2. Preparation of Spore Suspensions for Strain G59 and Its Mutant 5-1-4

Fresh spores formed by cultivation of strain G59 on PDA (potato dextrose agar) plates at 28 °C for 3–4 days were harvested, suspended in the appropriate amount of sterilized, distilled water with several glass beads in 50 mL cone-shaped flasks, and shaken well to prepare a crude spore suspension. A 100 μL portion of this crude spore suspension was added into a well of 96-well plates, diluted with water with its OD at 600 nm measured using a VERSAmax-BN03152 plate reader, and the dilution ratio was recorded when the OD value reached 0.35. Then, the whole crude spore suspension remaining was diluted with sterilized, distilled water in the same proportion to obtain a G59 spore suspension. This G59 spore suspension was used in the following experiments to keep the same spore density in all experiments concerned. The spore suspension for mutant 5-1-4, a gentamicin-resistant one, obtained by treatment of G59 with 5 mg/mL gentamicin in 50% (v/v) DMSO at 4 °C for 1 d (Table 3), was prepared in the same way as mentioned above for G59.

### 4.3. Mutant Selection by Treatment of G59 Spores with Gentamycin in Aqueous DMSO

To nine of the sterilized 2 mL Eppendorf tubes was added 0, 1, 2, 4, 10, 20, 40, 100 and 200 mg of gentamicin, respectively, and then 1600 μL of G59 spore suspension was added to dissolve gentamicin by shaking. Then, 400 μL DMSO was added to each tube to obtain a series of spore suspensions in 20% (v/v) DMSO. The one without gentamicin was used as a control and the other eight with 0.5, 1, 2, 5, 10, 20, 50 and 100 mg/mL gentamicin were used as the test group. To each of nine sterilized 2 mL Eppendorf tubes was added 0, 1, 2, 4, 10, 20, 40, 100 and 200 mg of gentamicin, respectively, as controls without DMSO, and then 1600 μL of G59 spore suspension was added and diluted with 400 μL of sterilized, distilled water to obtain 4:1 diluted G59 spore suspensions with the same spore density as in 20% (v/v) DMSO, which contained 0, 0.5, 1, 2, 5, 10, 20, 50 and 100 mg/mL gentamicin, respectively.

Similarly, to each of the six sterilized 2 mL Eppendorf tubes was added 0, 2, 4, 10, 20 and 40 mg of gentamicin, respectively, and 1000 μL of G59 spore suspension was added to dissolve gentamicin. Then, 1000 μL DMSO was added to each tube to obtain a series of spore suspensions in 50% (v/v) DMSO. The one without gentamicin was used as a control and the other five with 1, 2, 5, 10 and 20 mg/mL gentamicin were used as the test group in 50% (v/v) DMSO. Similarly, the 1:1 water diluted G59 spore suspensions containing 0, 1, 2, 5, 10 and 20 mg/mL gentamicin were used as the controls without DMSO, having the same spore density as in 50% (v/v) DMSO.

G59 spores in the above 20% (v/v) and 50% (v/v) DMSO tubes were treated at 4 °C for 1–20 days along with the controls in water. Each 90 μL of the treated spore suspensions was spread on PDA plates with a 24 h interval and incubated at 28 °C for 3–4 days. The mutant strains from the gentamicin-treatment test groups in 20% (v/v) and 50% (v/v) DMSO were obtained by single colony isolation, selecting colonies with different appearances during the incubation period.

### 4.4. Resistance Test for Acquired Resistance of Mutant 5-1-4 to Gentamicin

The 5-1-4 and G59 spore suspensions with 5 mg/mL gentamicin in 50% DMSO at the same spore density were prepared according to the procedure described in Sections 4.2 and Sections 4.3. The suspensions were stored at the same 4 °C condition for 1 day to treat the spores with gentamicin in the manner as that for selecting the mutant 5-1-4. Then, each 100 μL of the treated spore suspensions was spread on PDA plates, incubated at 28 °C, and examined day-by-day for growth up to 10 days of incubation. 

### 4.5. Fermentation and Sample Preparation for MTT Assay and Chemical Analysis

G59 and its mutants were inoculated into a 500 mL cone-shaped flask containing 200 mL of liquid medium (glucose 2%, maltose 1%, mannitol 2%, glutamic acid 1%, peptone 0.5% and yeast extract 0.3% in distilled water, adjusted to pH 6.0 prior to sterilization) and fermented at 28 °C for 13 days on a rotary shaker at 180 rpm. After fermentation, 200 mL broth was extracted with 400 mL acetone by ultrasonication for 1.5 h. The aqueous acetone solution obtained by filtration was concentrated under reduced pressure to remove acetone. The remaining water layer was then extracted three times with equal volumes of EtOAc to obtain the crude extract for MTT assay and chemical analysis (TLC, HPLC).

### 4.6. MTT Assay

The EtOAc extracts and pure compounds were dissolved in MeOH to prepare 10.0 mg/mL stock solutions, and serial dilutions were made for MTT assay. Exponentially growing K562 cells were suspended in fresh RPMI-1640 medium at the density of 1 × 10^5^ cells/mL, seeded into 96-well plates, and incubated at 37 °C for 1 h. Then 2 μL of MeOH for control or sample solutions was added to each well and the cells were cultured at 37 °C for 24 h. MTT (20 μL; 5 mg/mL in PBS) was added into each well, incubated at 37 °C for 4 h, and centrifuged at 2000 rpm for 20 min. After removal of the supernatant by aspirating, 150 μL DMSO was added into each well, and shaken for 5 min to dissolve formazan crystals. The OD value at 570 nm was read for each well using the VERSAmax-BN03152 plate reader. Each three wells were set for control and test groups, respectively, and the inhibition rate (IR%) was calculated using the formula, IR% = (OD_control_ − OD_sample_)/OD_control_ × 100%. The IC_50_ value for a sample was obtained from the IR% values of the sample at different concentrations.

### 4.7. TLC and HPLC Analysis

TLC analysis was carried out on precoated silica gel GF254 plates using CHCl_3_-MeOH (20:1, 15:1, 9:1 or 5:1), CH_2_Cl_2_-MeOH (15:1, 12:1, 9:1 or 5:1) or bp 60–90 °C Pet.Ether-Acetone (10:1, 5:1 or 1:1) as developing solvents. TLC spots were detected under UV light (254 and 365 nm) or by using iodine, Vaughan’s reagent [24 g of ammonium molybdate tetrahydrate (NH_4_)_6_Mo_7_O_24_·4H_2_O) and 1 g of ceric sulfate Ce(SO_4_)_2_ dissolved in 500 mL of 10% H_2_SO_4_] or 10% sulfuric acid reagent. HPLC analysis was performed using an analytical Venusil MP C18 column (5 μm, 100 Å, 4.6 mm × 250 mm; Agela Technologies) on a Waters HPLC system equipped with Waters 600 controller, Waters 600 pump, Waters 2414 refractive index detector, Waters 2996 photodiode array (PDA) detector and Waters Empower™ software. Each 10 μL of sample solutions (8.3 mg/mL) in MeOH was injected into the column and eluted with MeOH–H_2_O in linear gradient (20% MeOH at initial time 0 min→100% MeOH at 60 min→100% MeOH at 90 min) as mobile phase (flow rate, 1 mL/min). The acquired photodiode array (PDA) data were processed by Empower PDA software to obtain targeted HPLC data.

### 4.8. Time Course Experiment for Mutant 5-1-4 Fermentation

Spore suspension of mutant 5-1-4 (2 mL) was inoculated into a 500 mL cone-shaped flask with 200 mL of liquid medium (glucose 2%, maltose 1%, mannitol 2%, glutamic acid 1%, peptone 0.5% and yeast extract 0.3% in distilled water, adjusted to pH 6.0 prior to sterilization) and fermented at 28 °C for 14 days on a rotary shaker at 180 rpm. Sampling was performed for a 15 mL portion with a 24 h interval from the third day of fermentation. After centrifugation of the sampled broth at 3000 rpm for 10 min, followed by measurement of the pH value of supernatant and the relative percentage (%) of mycelial volume, the whole broth (15 mL) was extracted with 30 mL acetone by ultrasonication for 1.5 h. The obtained aqueous acetone solution was concentrated under reduced pressure to remove acetone and the remaining water layer was extracted three times with equal volumes of EtOAc to obtain the extracts for assay.

### 4.9. Fermentation and Extract Preparation

Based on the result of the time course experiment mentioned, spore suspension of mutant 5-1-4 (2 mL) was inoculated into each of 245 cone-shaped 500 mL flasks with 200 mL of liquid medium (glucose 2%, maltose 1%, mannitol 2%, glutamic acid 1%, peptone 0.5% and yeast extract 0.3% in distilled water, adjusted to pH 6.0 prior to sterilization) and fermented at 28 °C for 12 days on a rotary shaker at 180 rpm. The whole broth (49 L) was filtrated to separate into a filtrate and a mycelial cake. The filtrate was extracted three times with equal volumes of EtOAc to give an extract (14.3 g). The mycelial cake was extracted with 3 L of acetone-water (2:1) by ultrasonication for 1.5 h and the aqueous acetone solution obtained by filtration was evaporated under reduced pressure to remove acetone. The water layer remained was extracted three times with equal volumes of EtOAc to give another extract (16.3 g). The combined EtOAc extracts (30.6 g) showed an inhibitory effect on K562 cells, with an IR% value of 42.8% at the 100 µg*/*mL, from which the antitumor secondary metabolites **1**–**4** were isolated.

G59 spore suspension (2 mL) was inoculated into each of two 500 mL cone-shaped flasks containing 200 mL of liquid medium with the same composition and fermented at 28 °C for 12 days under the same conditions. The fermented broth (400 mL) was extracted as described above for mutant 5-1-4 to obtain an EtOAc extract (200 mg), which did not show any inhibitory effect on K562 cells (an IR% value of 6.0% at 100 µg*/*mL). This extract was used as negative control in the experiments for tracing antitumor secondary metabolites newly produced by the mutant 5-1-4.

### 4.10. Isolation of Antitumor Metabolites 1–4 Produced by the Mutant 5-1-4

The EtOAc extract (30.6 g) of mutant 5-1-4 was separated into 15 fractions (**Fr**-**1** to **Fr**-**15**) by vacuum liquid chromatography (VLC) using a silica gel column (bed 6.2 × 10 cm) dry-packed with 150 g of silica gel H (100–200 meshes) through stepwise elution with bp 60–90 °C petroleum ether (P)-ethyl acetate (E) (100:0→0:100) and then dichloromethane (D)-methanol (M) (90:10→0:100). Six fractions, **Fr**-**4** (1.6 g) eluted by PE (9:1→8:2), **Fr**-**5** (3.7 g) eluted by PE (8:2), **Fr**-**6** (2.0 g) eluted by PE (8:2→7:3), **Fr**-**7** (4.6 g) eluted by PE (7:3→4:6), **Fr**-**8** (2.5 g) eluted by PE (2:8) and **Fr**-**12** (5.1 g) eluted by DM (9:1→8:2), showed inhibitory effects against K562 cells, with IR% values of 34.4, 31.4 36.5, 52.8, 43.0, and 49.4%, respectively, at 100 μg/mL.

**Fr**-**4** (1.6 g) was subsequently subjected to Sephadex LH-20 column (bed 1.5 × 135 cm in DM 1:1) chromatography using DM (1:1) as eluting solvent to obtain seven fractions, **Fr**-**4**-**1** to **Fr**-**4**-**7**, in the order of elution. **Fr**-**4**-**3** (1.45 g) was further separated by VLC using a silica gel column (bed 2.5 × 6 cm) dry-packed with 12 g of silica gel H (200–300 meshes) by a stepwise elution with PE (100:0→0:100) and EM (100:0→0:100) to give seven fractions, **Fr**-**4**-**3**-**1** to **Fr**-**4**-**3**-**7**. Among them, **Fr**-**4**-**3**-**2** (420 mg) eluted by PE (99:1→97:3) gave yellow crude crystals at room temperature, which were filtered and then repeatedly recrystallized from dichloromethane–methanol (1:1) to obtain **1** (28 mg) as yellowish needles.

**Fr**-**5** (3.7 g) was further separated into 12 fractions, **Fr**-**5**-**1** to **Fr**-**5**-**12**, by VLC on a silica gel column (bed 2 × 12 cm) dry-packed with 25 g of silica gel H (100–200 meshes) through stepwise elution with PE (100:0→0:100) and DM (90:10→0:100). **Fr**-**5**-**2** (160 mg) eluted by PE (99:1→96:4) was separated by Sephadex LH-20 column (bed 1.5 × 135 cm in DM 1:1) chromatography eluted with DM (1:1) to obtain five fractions, **Fr**-**5**-**2**-**1** to **Fr**-**5**-**2**-**5**, in the order of elution. **Fr**-**5**-**2**-**1** (70 mg) was separated by semi-preparative HPLC on a reversed-phase Senshu Pak C18 column (8 × 250 mm) using 65% aqueous MeOH as eluting solvent (flow rate 1.5 mL/min, detector wave length 246 nm) to obtain a fraction corresponding to the peak with a retention time (t*_R_*) 38 min. This fraction was recrystallized in dichloromethane to give **2** (30 mg) as colorless needles.

**Fr**-**6** (2.0 g) was also further separated by Sephadex LH-20 column (bed 1.5 × 135 cm in DM 1:1) chromatography using DM (1:1) as eluents to give seven fractions, **Fr**-**6**-**1** to **Fr**-**6**-**7**, in the order of elution. **Fr**-**6**-**4** (490 mg) was separated by preparative TLC on silica gel GF254 plates (0.5 mm thickness, 20 × 20 mm) using cyclohexane-acetone-ammonia water (30:10:1) as the developing solvents. The silica gel on the band with the *R*_f_ value of 0.6 under 254 nm UV light was collected to afford **3** (8 mg) as colorless oil from MeOH solution.

**Fr**-**8** (2.5 g) gave yellow crude needles at room temperature in MeOH. The crystals were filtered and repeatedly recrystallized from MeOH to obtain **4** (40 mg) as lemon-yellow needles.

### 4.11. Physicochemical and Spectral Data for Compounds ***1–4***

Janthinone (**1**): yellowish needles (CH_2_Cl_2_-MeOH), mp 197–198 °C. Positive ESI-MS *m/z*: 285 [M + H]^+^, 307 [M + Na]^+^. ^1^H NMR (400 MHz, CDCl_3_) δ: 12.06 (1H, s, 8-OH), 7.65 (1H, dd, *J = *8.5, 7.3 Hz, H-3), 7.43 (1H, dd, *J = *8.5, 1.0 Hz, H-4), 7.22 (1H, dd, *J = *7.3, 1.0 Hz, H-2), 6.65 (1H, br s, H-5), 6.53 (1H, br s, H-7), 3.95 (3H, s, H_3_-11), 2.34 (3H, s, H_3_-12). ^13^C NMR (100 MHz, CDCl_3_) δ: 180.4 (C-9), 169.6 (C-10), 161.4 (C-8), 155.9 (C-4a), 155.6 (C-4b), 149.3 (C-6), 134.7 (C-3), 133.5 (C-1), 122.4 (C-2), 119.4 (C-4), 117.5 (C-9a), 111.6 (C-7), 107.3 (C-5), 106.9 (C-8a), 53.1 (C-11), 22.6 (C-12). The above data for **1** are identical with those reported for janthinone [51].

Fructigenine A (**2**): colorless needles (CH_2_Cl_2_), mp 102–105 °C, [α]^20^_D_ −175.4 (*c* 0.24, CHCl_3_). Positive ESI-MS *m/z*: 444 [M + H]^+^; negative ESI-MS *m/z*: 442 [M − H]^−^. ^1^H NMR (400 MHz, CDCl_3_) δ: 7.94 (1H, br s, H-7), 7.30–7.11 (7H, m, H-8,10,14,15,16,17,18), 7.06 (1H, td, *J* = 7.6, 1.1 Hz, H-9), 5.98 (1H, br s, H-2), 5.70 (1H, dd, *J *= 17.6, 10.8 Hz, H-20), 5.57(1H, br s, H-5a), 5.06 (1H, d, *J *= 10.8 Hz, H*cis*-21), 5.04 (1H, d, *J *= 17.6 Hz, H*trans*-21), 4.16 (1H, dd, *J *= 10.4, 2.8 Hz, H-3), 3.73 (1H, dd, *J *= 11.7, 5.5 Hz, H-11a), 3.48 (1H, dd, *J *= 14.4, 2.8 Hz, Ha-12), 2.74 (1H, dd, *J *= 14.4, 10.4 Hz, Hb-12), 2.59 (3H, s, H_3_-23), 2.50 (1H, dd, *J *= 11.7, 5.5 Hz, Ha-11), 2.20 (1H, t, *J *= 11.7 Hz, Hb-11), 1.06 (3H, s, H_3_-19b), 0.91 (3H, s, H_3_-19a). ^13^C NMR (100 MHz, CDCl_3_) δ: 170.1 (C-22), 168.1 (C-4), 164.8 (C-1), 143.3 (C-7a), 143.0 (C-20) , 135.3 (C-13), 131.7 (C-10a), 129.4 (2C, C-15,17), 129.1 (3C, C-8,14,18), 127.7 (C-16), 124.6 (2C, C-7,10), 119.1 (C-9), 114.6 (C-21), 79.5 (C-5a), 60.9 (C-10b), 59.1 (C-11a), 56.0 (C-3), 40.4 (C-19), 37.0 (C-11), 36.0 (C-12), 23.6 (C-19b), 23.2 (C-19a), 22.4 (C-23). The above data for **2** are identical with those of fructigenine A [52].

Aspterric acid methyl ester (**3**): colorless oil (MeOH), [α]^20^_D_ −59 (*c* 1.0, MeOH), molecular formula C_16_H_24_O_4_. Positive ESI-MS *m/z*: 281 [M + H]^+^, 303 [M + Na]^+^. Positive HR-ESI-MS *m/z*: measured 281.1757 [M + H]^+^, calculated for C_16_H_25_O_4_ [M + H] 281.1753. ^1^H NMR (400 MHz, CD_3_OD) δ: 4.31 (1H, d, *J *= 8.4 Hz, H-2), 3.71 (3H, s, H_3_-16), 3.67 (1H, br d, *J = *8.2 Hz, Ha-13, gave correlation peak with H-6 at δ2.31 in ^1^H–^1^H COSY), 3.34 (1H, dd, *J = *8.2, 1.2 Hz, Hb-13, gave correlation peak with H-6 at δ2.31 in ^1^H–^1^H COSY), 2.37–2.45 (2H, m, Ha-4/Ha-5), 2.32 (1H, br d, *J = *9.4 Hz, H-6, gave correlation peaks with Ha-13 at δ3.67 and Hb-13 at δ3.34 in ^1^H-^1^H COSY) 2.25–2.35 (1H, m, Ha-9), 2.16 (1H, dd, *J = *13.2, 8.4 Hz, Ha-1), 2.01 (1H, d, *J = *13.2 Hz, Hb-1), 1.96–2.07 (1H, m, Hb-9), 1.73 (3H, s, H_3_-14), 1.71 (1H, td, *J = *12.0, 3.9 Hz, Ha-8), 1.62–1.67 (1H, m, Hb-5), 1.60 (3H, s, H_3_-15), 1.49 (1H, dd, *J = *12.0, 7.5 Hz, Hb-8), 1.42 (1H, br t, *J = *12.5 Hz, Hb-4). ^13^C-NMR (100 MHz, CD_3_OD) δ: 176.2 (C-12), 136.6 (C-10), 125.2 (C-11), 84.9 (C-2), 79.8 (C-3), 76.5 (C-13), 56.7 (C-6), 54.3 (C-7), 52.4 (C-16), 36.6 (C-1), 35.2 (C-4), 35.0 (C-8), 33.1 (C-9), 24.7 (C-5), 23.4 (C-15), 21.0 (C-14). The above NMR data for **3** assigned by the analysis of DEPT, ^1^H-^1^H COSY, HMQC and HMBC spectra closely resembled those of aspterric acid [53], except for additional signals due to a methoxy group (δ_H_3.72 and δ_C_52.4, H_3_-16 and C-16), leading to assignment of **3** as aspterric acid methyl ester.

Citrinin (**4**): lemon yellow needles (MeOH), mp 178–179 °C, [α]^20^_D_ −17.3 (*c* 1.0, EtOH). Positive ESI-MS *m/z*: 251 [M + H]^+^, 273 [M + Na]^+^, 289 [M + K]^+^. ^1^H NMR (400 MHz, CDCl_3_) δ: 15.91 (1H, s, 12-OH), 15.13 (1H, s, 8-OH), 8.25 (1H, s, H-1), 4.79 (1H, q, *J = *6.8 Hz, H-3), 3.00 (1H, q, *J = *7.0 Hz, H-4), 2.02 (3H, s, H_3_-11), 1.35 (3H, d, *J = *6.8 Hz, H_3_-9), 1.23 (3H, d, *J = *7.0 Hz, H_3_-10). ^13^C NMR (100 MHz, CDCl_3_) δ: 183.7 (C-6), 177.1 (C-8), 174.5 (C-12), 162.9 (C-1), 139.1 (C-4a), 123.0 (C-5), 107.3 (C-8a), 100.2 (C-7), 81.6 (C-3), 34.5 (C-4), 18.5 (C-11), 18.2 (C-9), 9.4 (C-10). The above data are identical with those reported for citrinin [54,55].

## 5. Conclusions

To summarize, a new approach to activate dormant secondary metabolite production has been developed by introducing gentamicin-resistance in *Penicillium purpurogenum* G59, which is inactive, to produce antitumor secondary metabolites. Treatment of the G59 spores with a high concentration of gentamicin in aqueous DMSO afforded a total of 181 mutants, nine of which acquired metabolic capability to produce antitumor secondary metabolites as indicated by the antitumor effects of their extracts. This was evidenced also by the TLC and HPLC analysis of extracts of G59 and the nine mutants. Identification of four newly produced antitumor metabolites by mutant 5-1-4, further demonstrated the effectiveness of this approach. The present study provides a simple and practical approach without a complex gene manipulation process to activate dormant secondary metabolite production in fungi, which could be applied to other fungal metabolite studies.

## Supplementary Files

Supplementary File 1: ZIP-Document (ZIP, 1158 KB)
