# Supplementary material for: Activation of the Dormant Secondary Metabolite Production by Introducing Gentamicin-Resistance in a Marine-Derived Penicillium purpurogenum G59
Source: Mar Drugs. 2012 Mar 2;10(3):559–82. doi: 10.3390/md10030559 (PMC3347015; doi:10.3390/md10030559)
Supplement: Supplementary File 1: — ZIP-Document (ZIP, 1158 KB) [file marinedrugs-10-00559-s001.zip › marinedrugs-13100-supplementary/marinedrugs-13100-Supplementary Data S2 - TLC and HPLC anyalysis for Compounds 1-7.pdf]

Activation of the dormant secondary metabolite production by introducing gentamicin-resistance in a marine-derived *Penicillium purpurogenum* G59

Yun-Jing Chai, Cheng-Bin Cui *, Chang-Wei Li, Chang-Jing Wu, Cong-Kui Tian and Wei Hua

Prof. Dr. C.-B. Cui, Beijing Institute of Pharmacology and Toxicology, 27 Tai-Ping Road, Haidian District, Beijing 100850, China. E-mail: cuicb@sohu.com, cuicb@126.com; Tel./Fax.: +86-10-6821- 1656.

Supplementary Data S2 – TLC and HPLC Analysis for Compounds 1-7

Contents of Supplementary Data S2

1. TLC analysis for compounds **1–4** and for **2** and **5** ....page S2-1

2. HPLC analysis for compounds **1**, **2** and **4–7** .... page S2-1

*2.1. HPLC analysis for **1** by comparison with G59 extract* ....page S2-2
*2.2. HPLC analysis for **2** by comparison with G59 extract* ....page S2-3
*2.3. HPLC analysis for **4** by comparison with G59 extract* ....page S2-5
*2.4. HPLC analysis for **5** by comparison with G59 extract* ....page S2-7
*2.5. HPLC analysis for **6** by comparison with G59 extract* ....page S2-9
*2.6. HPLC analysis for **7** by comparison with G59 extract* ....page S2-11

1. TLC analysis for compounds **1–4** and for **2** and **5**

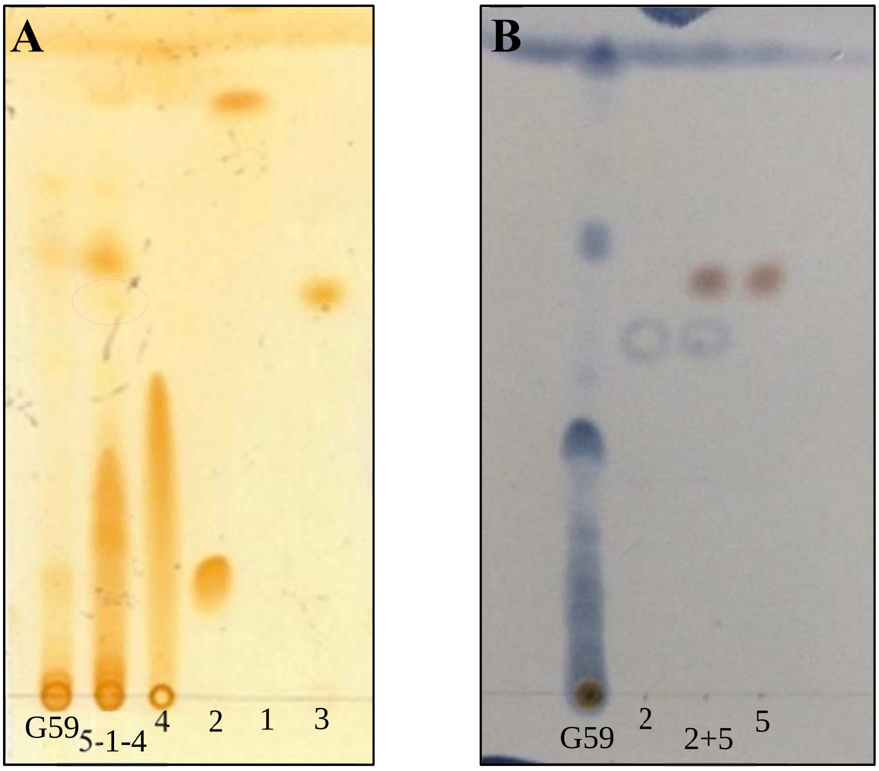

Silica Gel GF254 Plate TLC Chromatograms for Compounds **1–5**
Developing solvent: **A**, CH_2Cl_2–Acetone 5:1; **B**, $CHCl_3$–MeOH 30:1
Detecting reagent: **A**, I (iodine); **B**, Vaughan's reagent

2. HPLC analysis for compounds **1**, **2** and **4–7**

*2.1 HPLC analysis for compound **1** by comparison with the EtOAc extract of G59 culture*

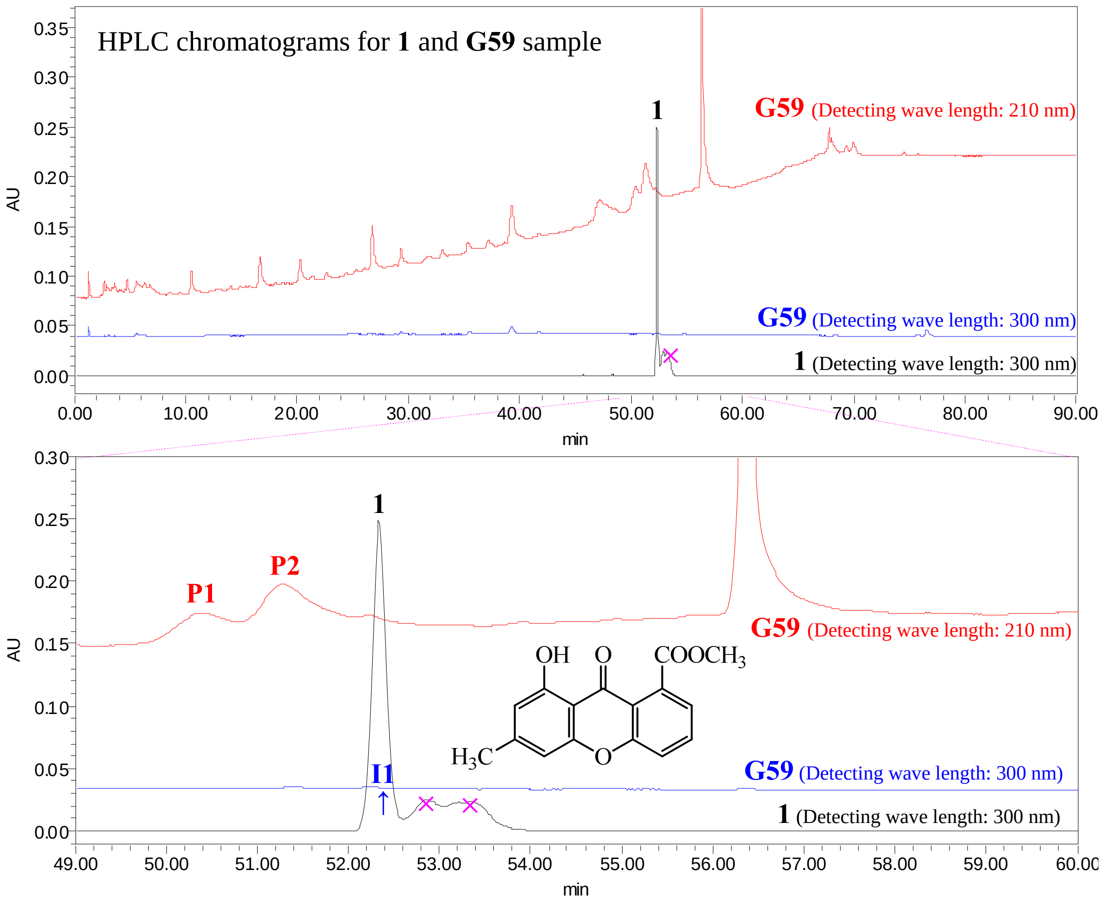

Normalized HPLC chromatograms for **1** and **G59** sample

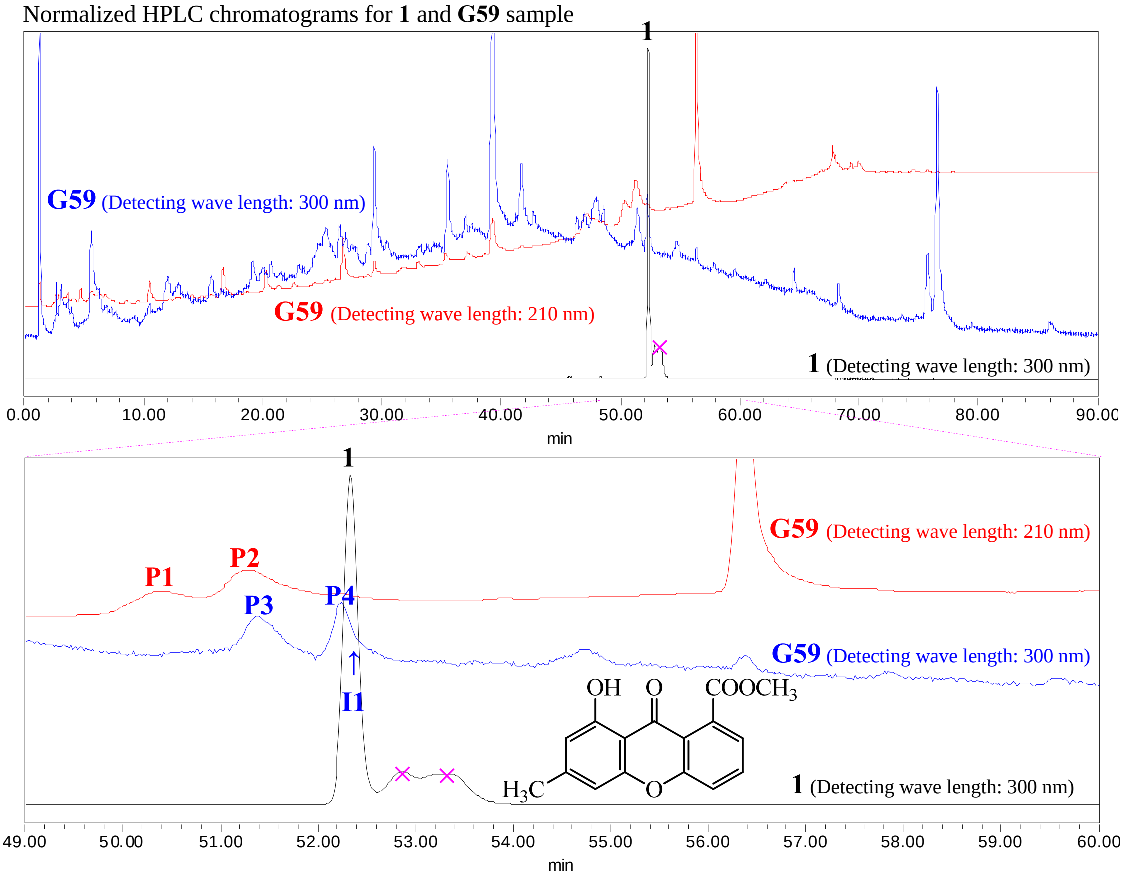

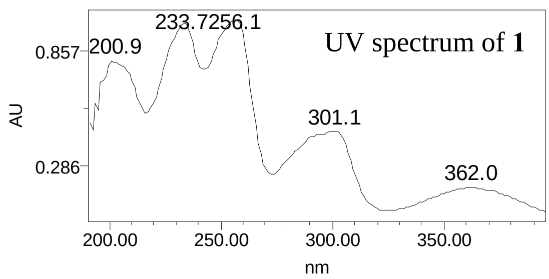

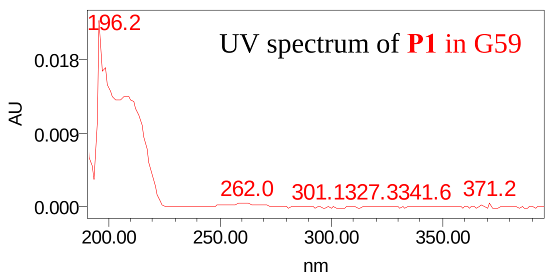

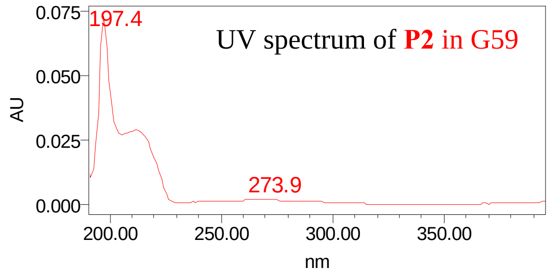

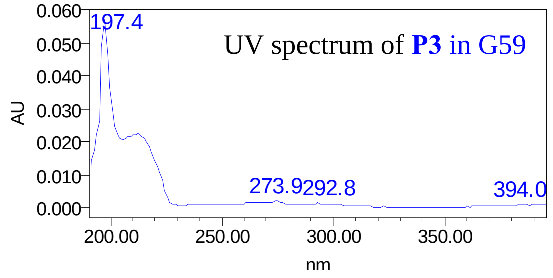

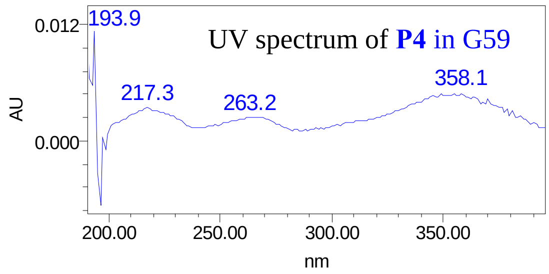

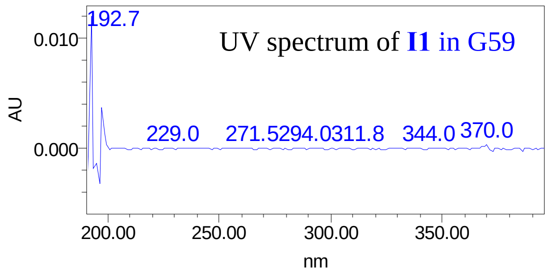

*2.2 HPLC analysis for compound **2** by comparison with the EtOAc extract of G59 culture*

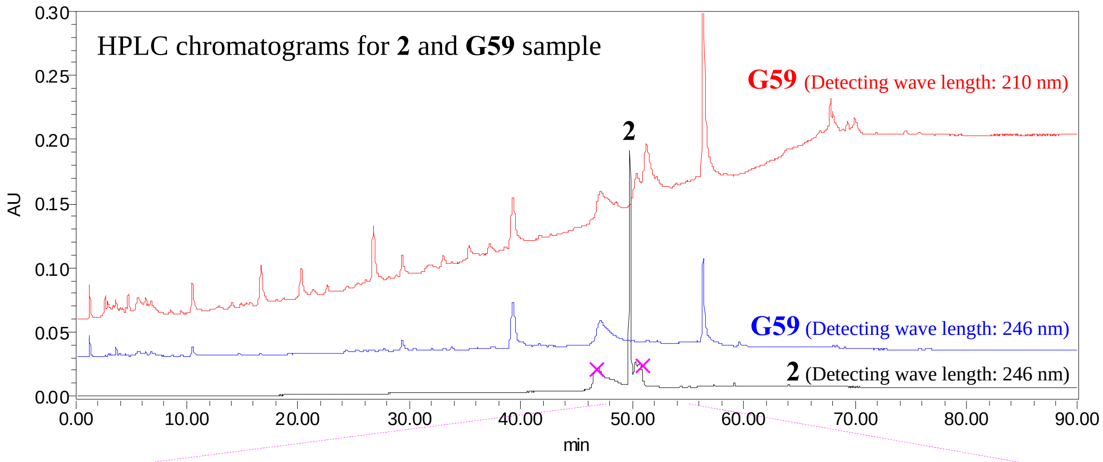

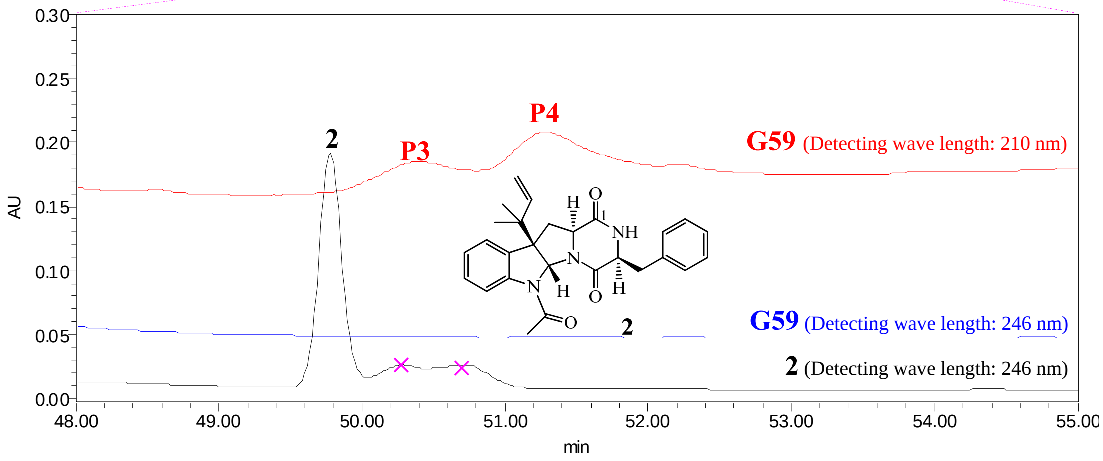

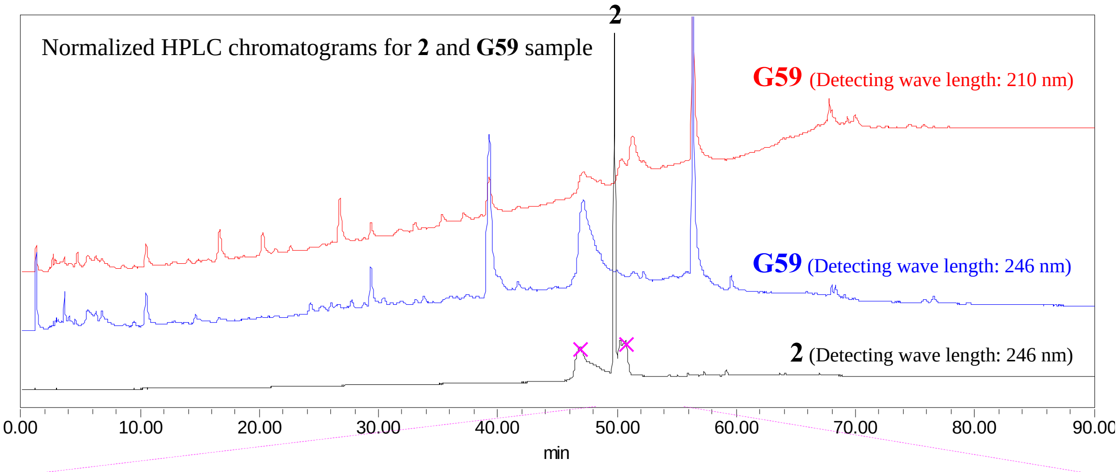
Normalized HPLC chromatograms for 2 and G59 sample
2
G59 (Detecting wave length: 210 nm)
G59 (Detecting wave length: 246 nm)
2 (Detecting wave length: 246 nm)
0.00 10.00 20.00 30.00 40.00 50.00 60.00 70.00 80.00 90.00
min

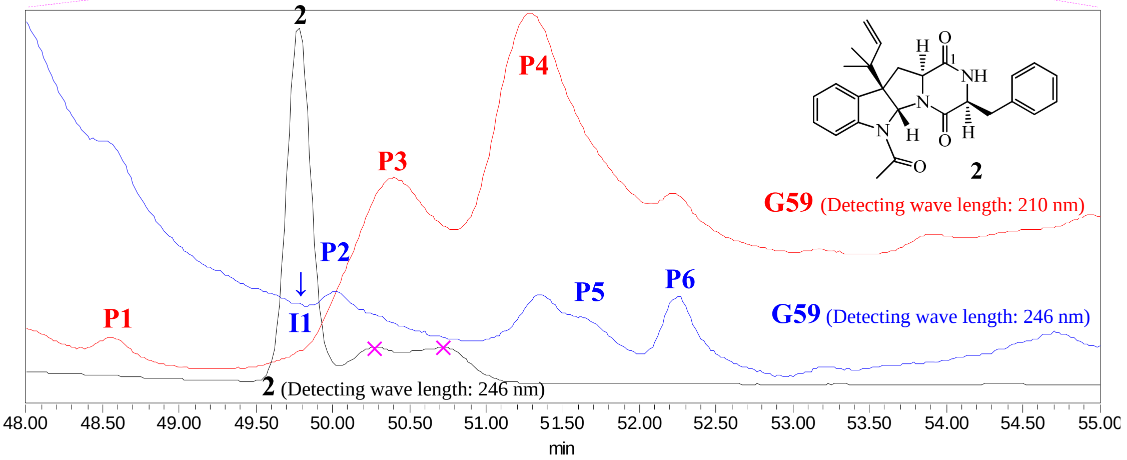
2
P4
P3
P2
I1
P1
P5
P6
G59 (Detecting wave length: 210 nm)
G59 (Detecting wave length: 246 nm)
2 (Detecting wave length: 246 nm)
H
O
NH
N
N
H
O
H
O
2
48.00 48.50 49.00 49.50 50.00 50.50 51.00 51.50 52.00 52.50 53.00 53.50 54.00 54.50 55.00
min

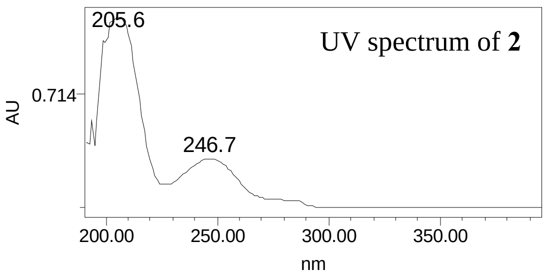
UV spectrum of 2
205.6
246.7
0.714
AU
200.00 250.00 300.00 350.00
nm

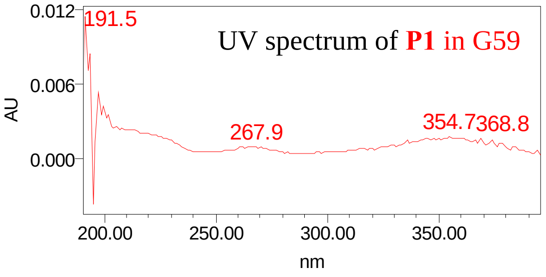
UV spectrum of P1 in G59
191.5
267.9
354.7368.8
0.012
0.006
0.000
AU
200.00 250.00 300.00 350.00
nm

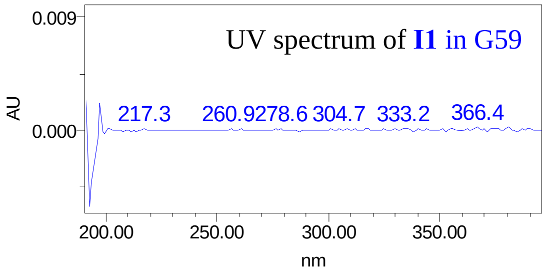
UV spectrum of I1 in G59
217.3 260.9278.6 304.7 333.2 366.4
0.009
0.000
AU
200.00 250.00 300.00 350.00
nm

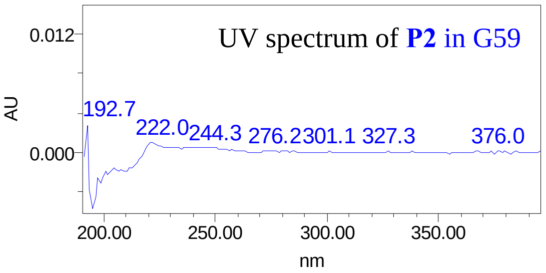
UV spectrum of P2 in G59
192.7
222.0244.3 276.2301.1 327.3 376.0
0.012
0.000
AU
200.00 250.00 300.00 350.00
nm

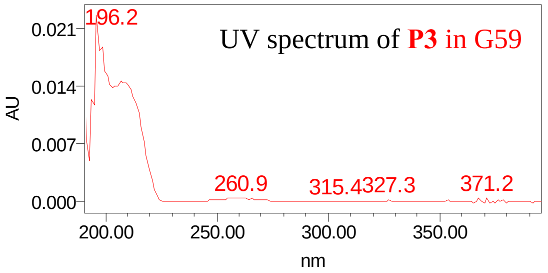
UV spectrum of P3 in G59
196.2
260.9
315.4327.3
371.2
0.021
0.014
0.007
0.000
AU
200.00 250.00 300.00 350.00
nm

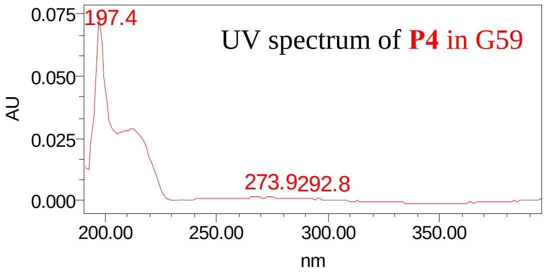
UV spectrum of P4 in G59
197.4
273.9292.8
0.075
0.050
0.025
0.000
AU
200.00 250.00 300.00 350.00
nm

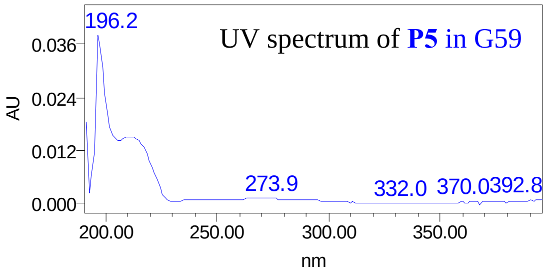

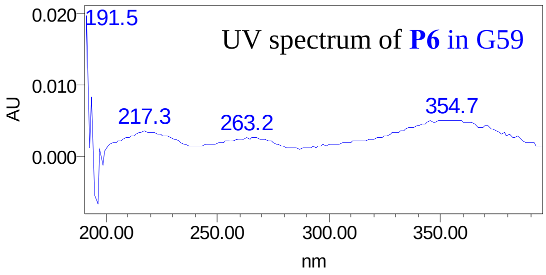

*2.3 HPLC analysis for compound **4** by comparison with the EtOAc extract of G59 culture*

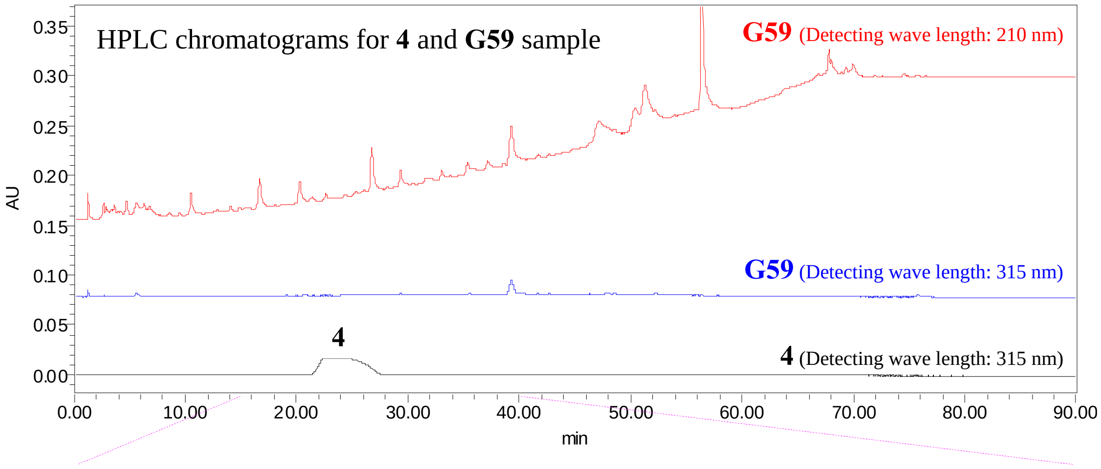

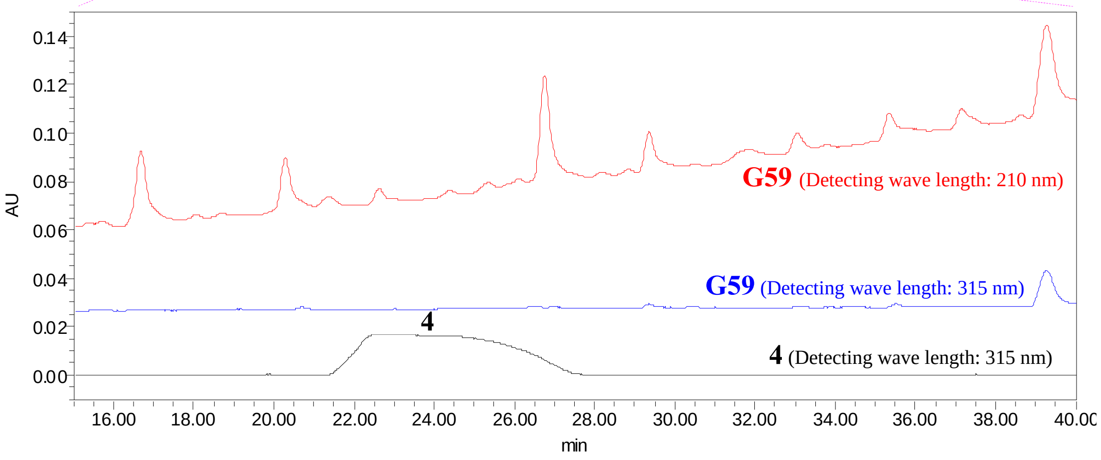

Normalized HPLC chromatograms for **4** and G59 sample

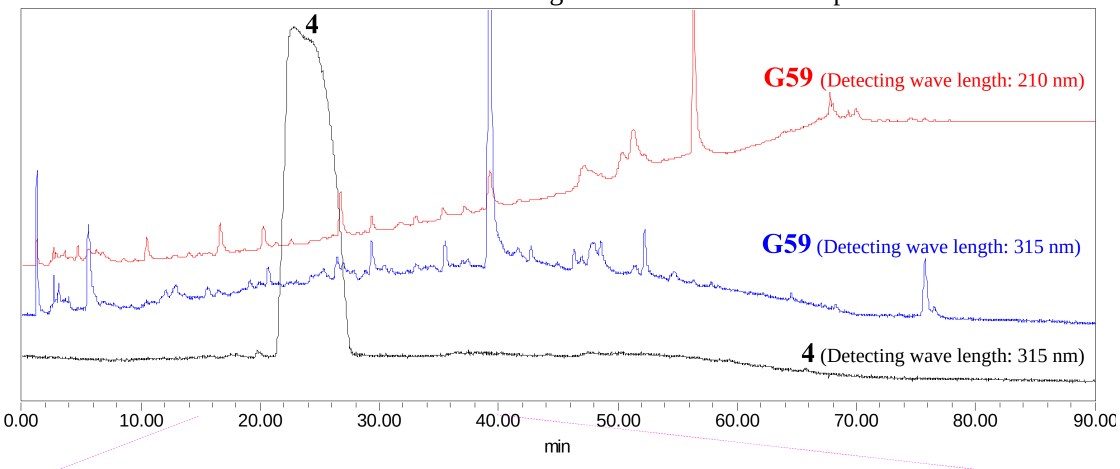

Expanded chromatograms see in next page

Expansion of normalized HPLC chromatograms for **4** and **G59** sample

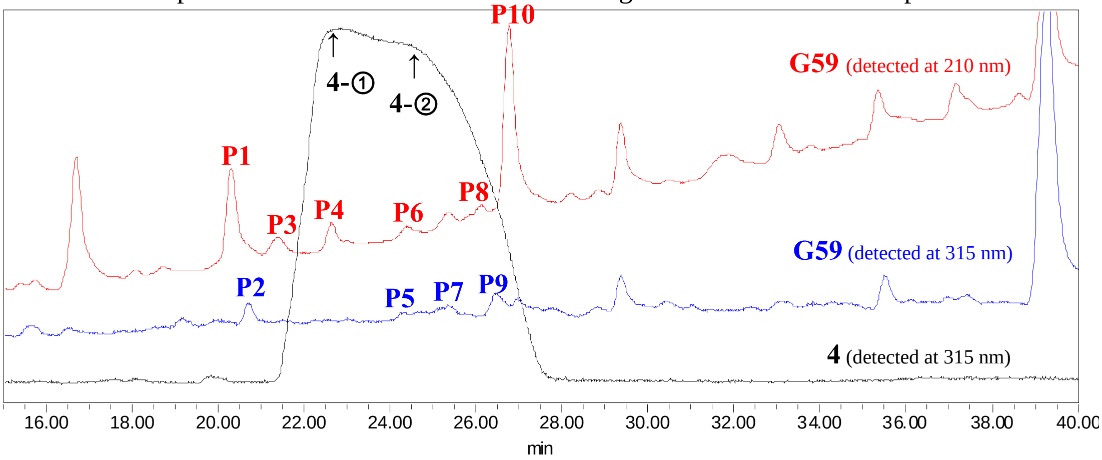

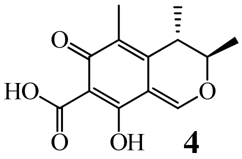

4

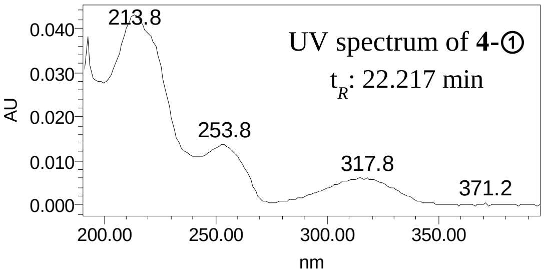

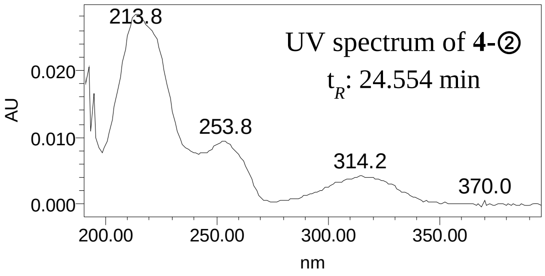

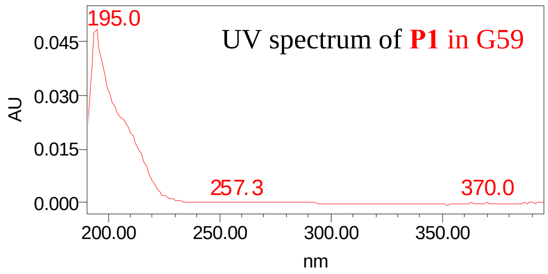

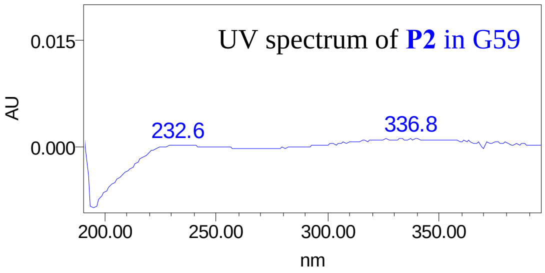

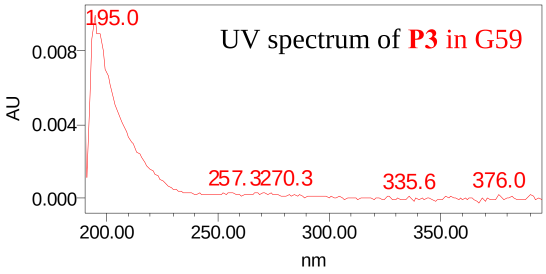

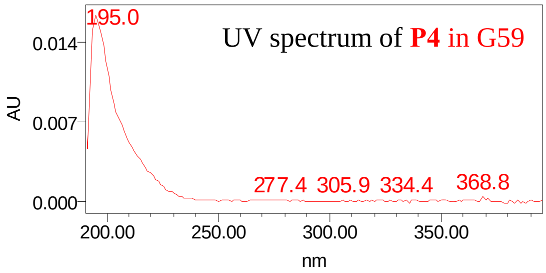

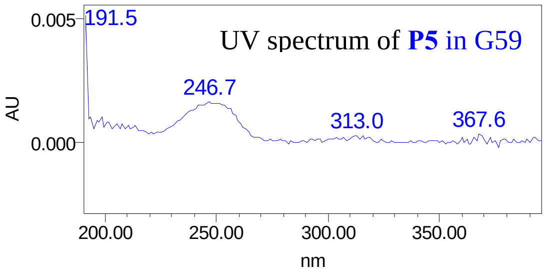

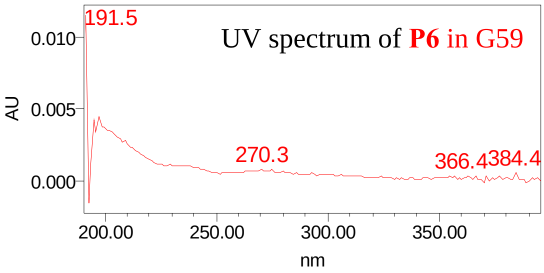

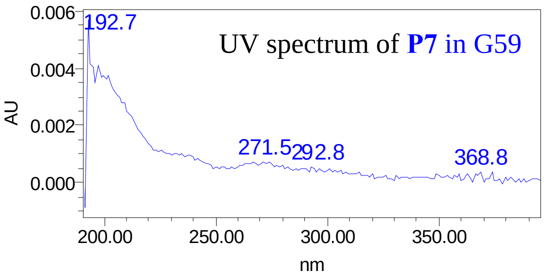

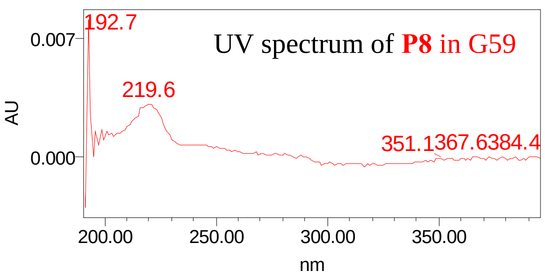

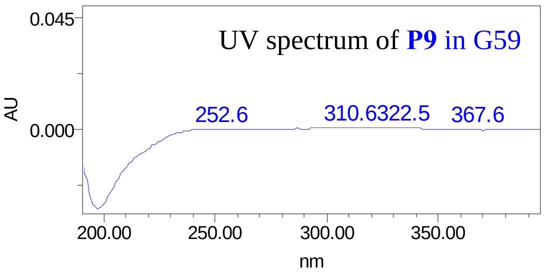

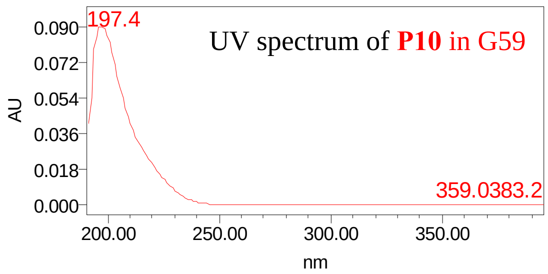

*2.4 HPLC analysis for compound **5** by comparison with the EtOAc extract of G59 culture*

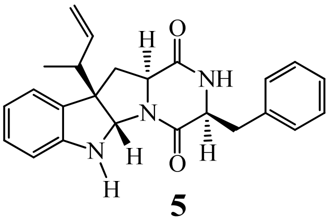

5

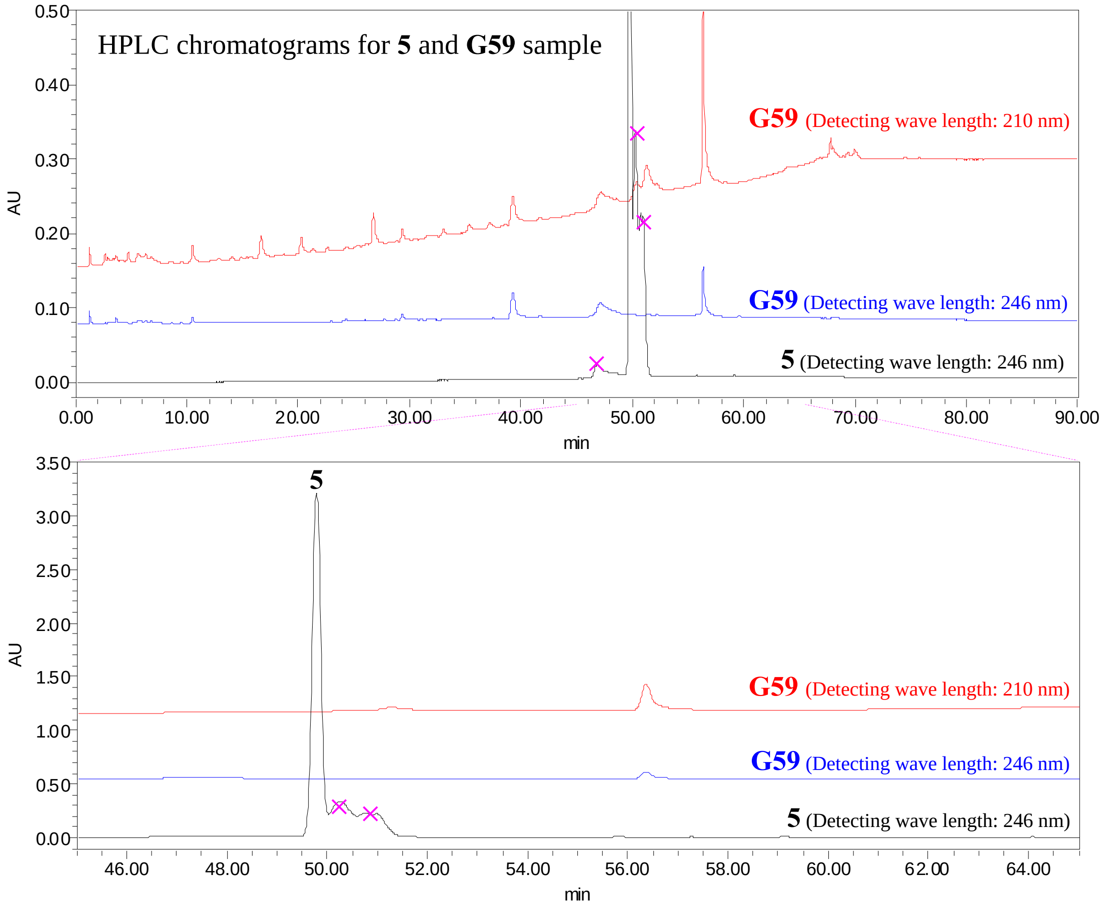

Normalized HPLC chromatograms for **5** and **G59** sample

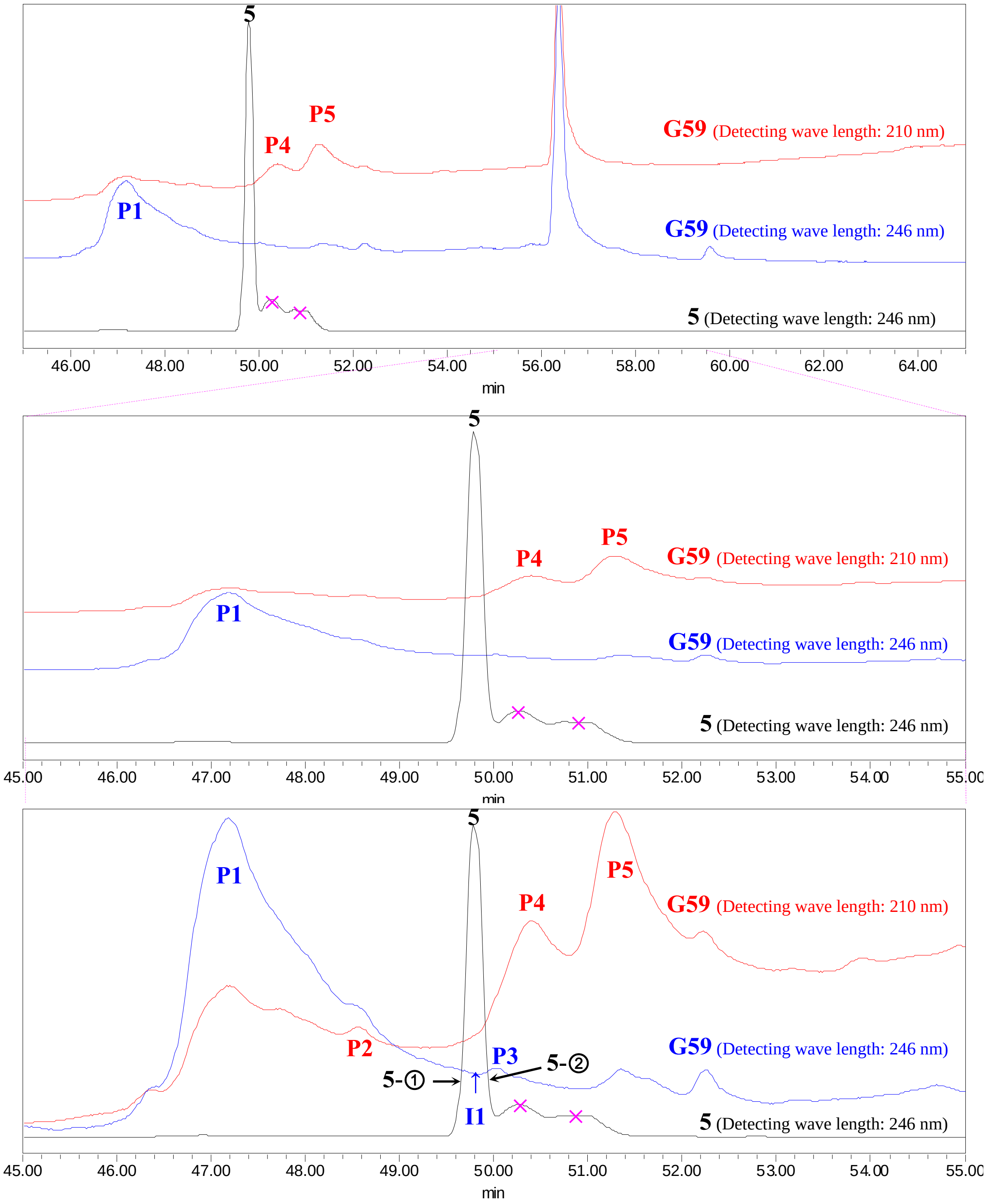

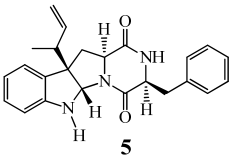

5

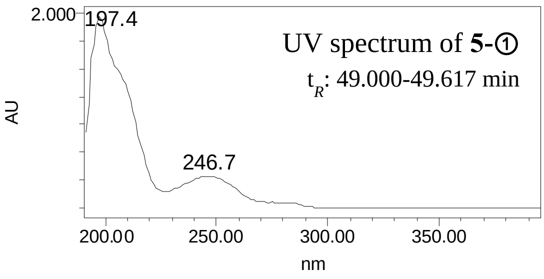

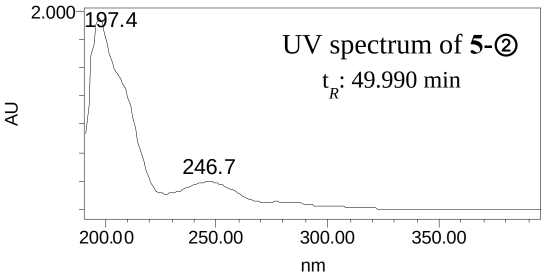

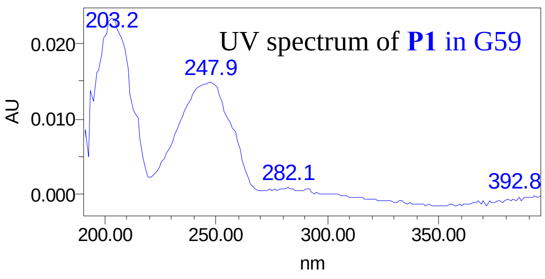

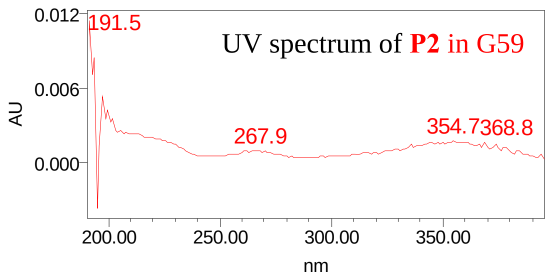

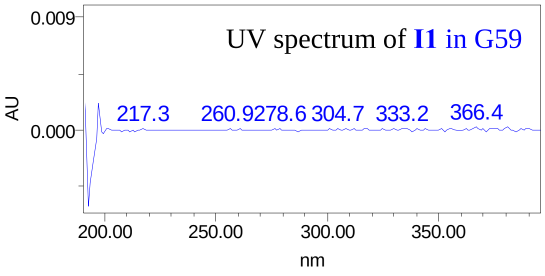

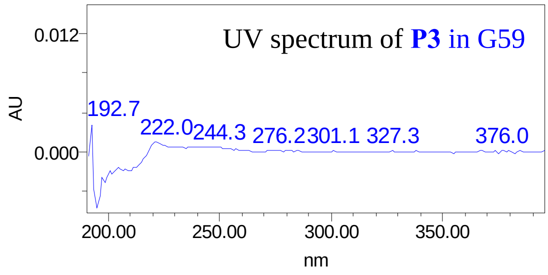

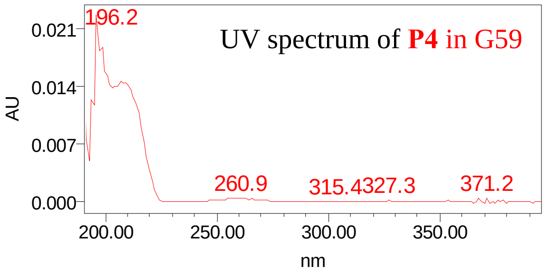

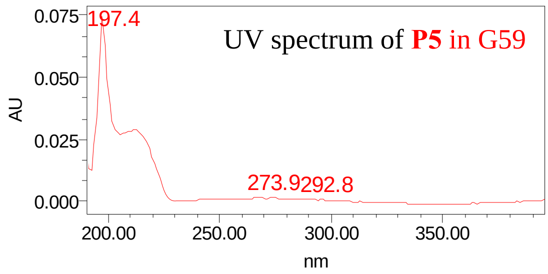

*2.5 HPLC analysis for compound **6** by comparison with the EtOAc extract of G59 culture*

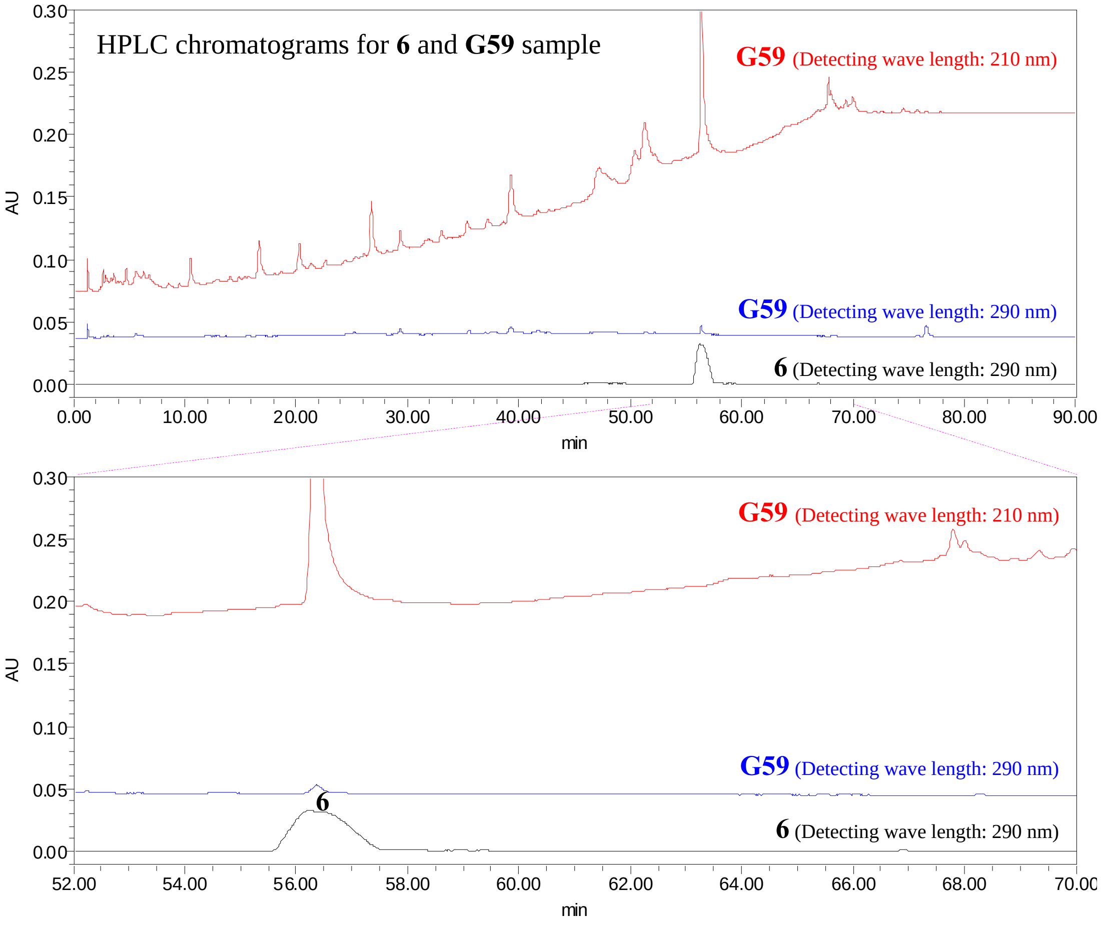

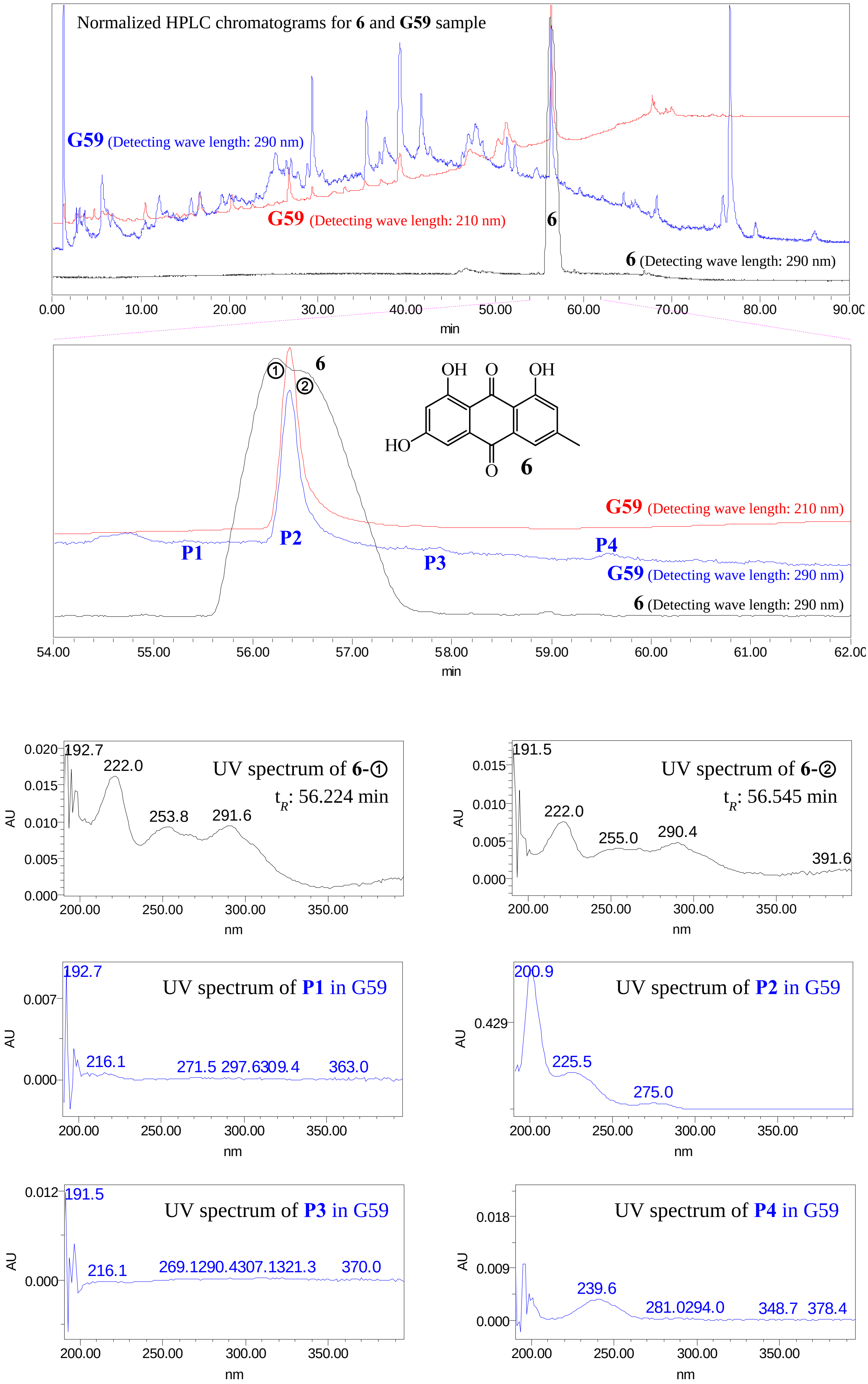
Normalized HPLC chromatograms for 6 and G59 sample
G59 (Detecting wave length: 290 nm)
G59 (Detecting wave length: 210 nm)
6
6 (Detecting wave length: 290 nm)
min
①
②
6
OH
O
OH
HO
O
6
G59 (Detecting wave length: 210 nm)
P1
P2
P3
P4
G59 (Detecting wave length: 290 nm)
6 (Detecting wave length: 290 nm)
min
UV spectrum of 6-①
tR: 56.224 min
192.7
222.0
253.8
291.6
AU
nm
UV spectrum of 6-②
tR: 56.545 min
191.5
222.0
255.0
290.4
391.6
UV spectrum of P1 in G59
192.7
216.1
271.5 297.6309.4
363.0
UV spectrum of P2 in G59
200.9
225.5
275.0
UV spectrum of P3 in G59
191.5
216.1
269.1290.4307.1321.3
370.0
UV spectrum of P4 in G59
239.6
281.0294.0
348.7
378.4

*2.6 HPLC analysis for compound **7** by comparison with the EtOAc extract of G59 culture*

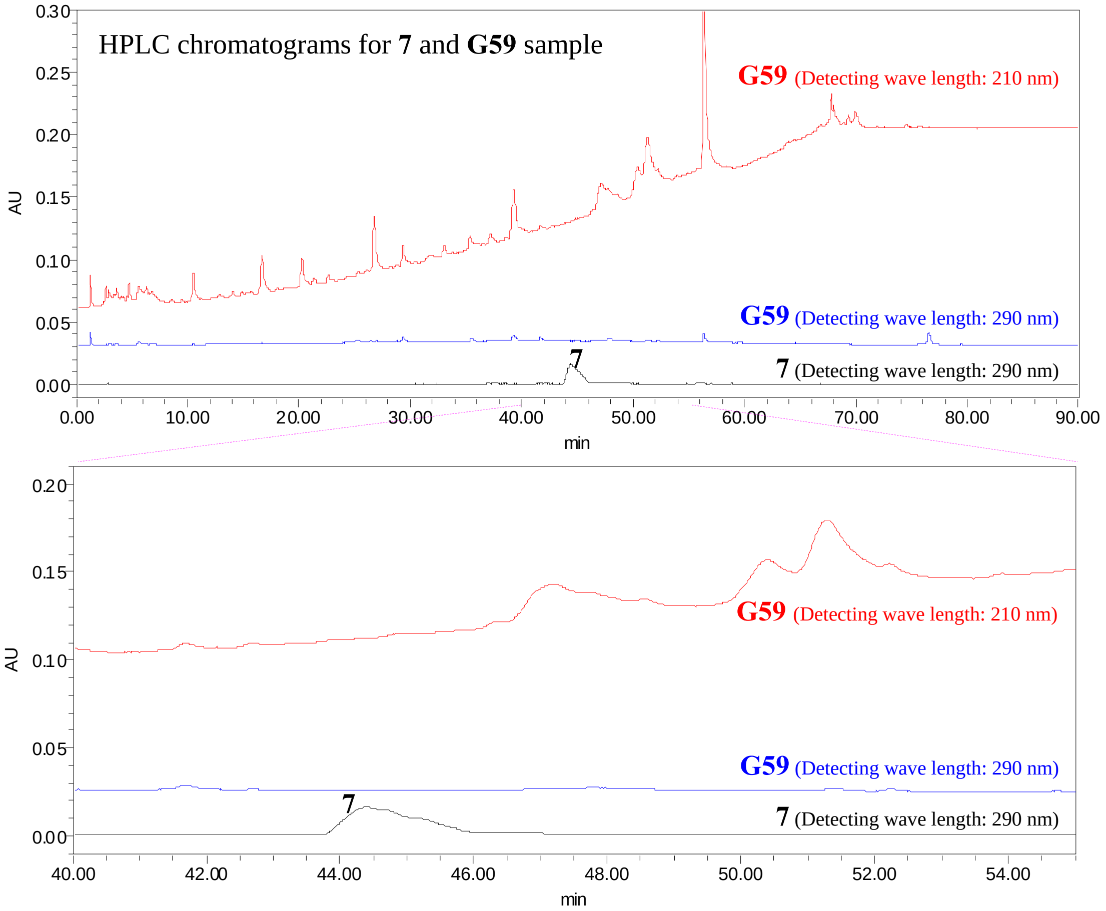

Normalized HPLC chromatograms for **7** and **G59** sample

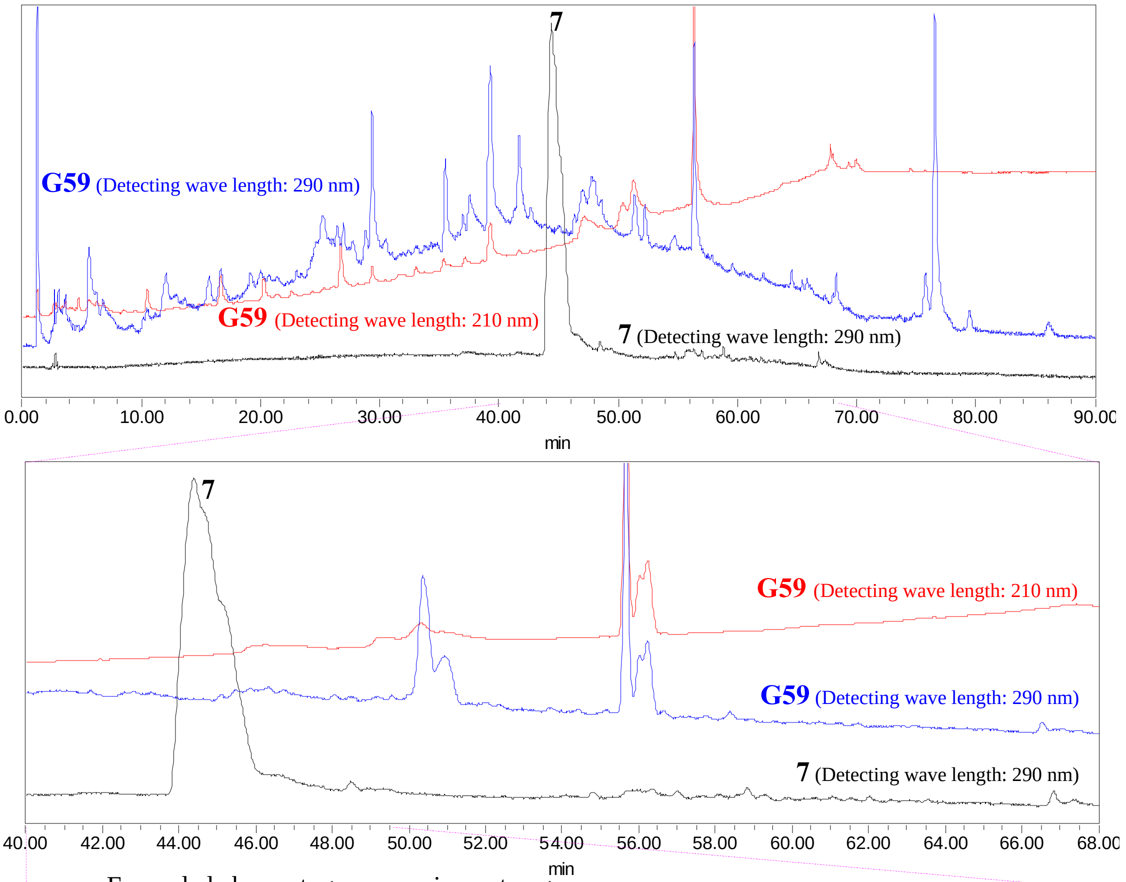

Expanded chromatograms see in next page

Expansion of normalized HPLC chromatograms for **7** and **G59** sample

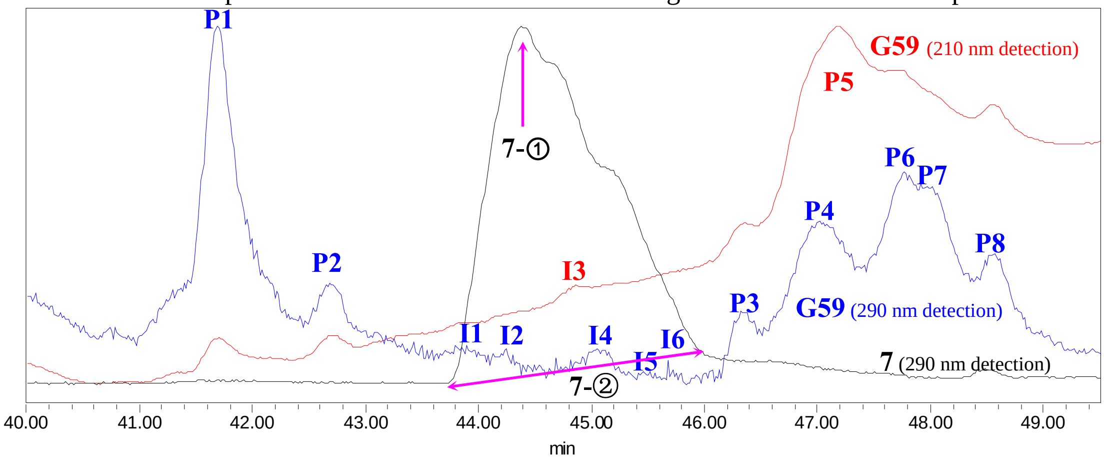

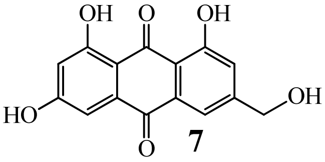

7

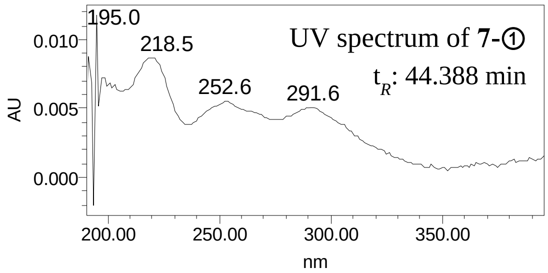

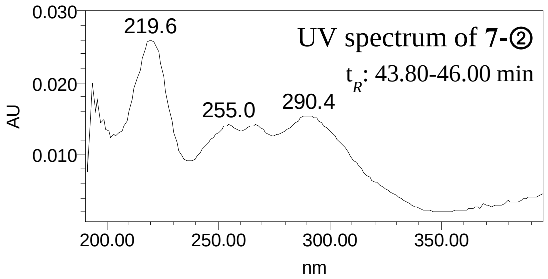

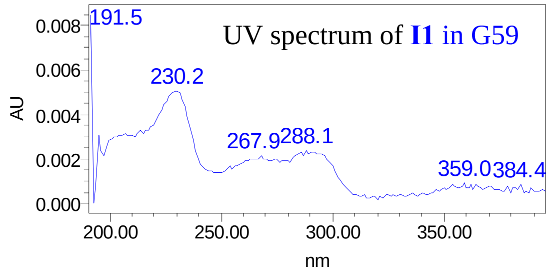

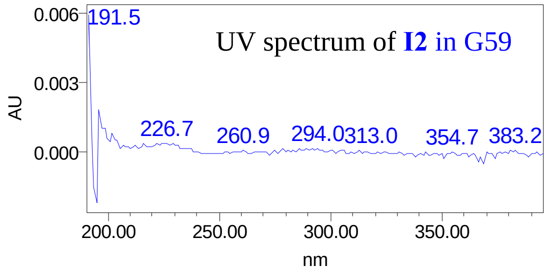

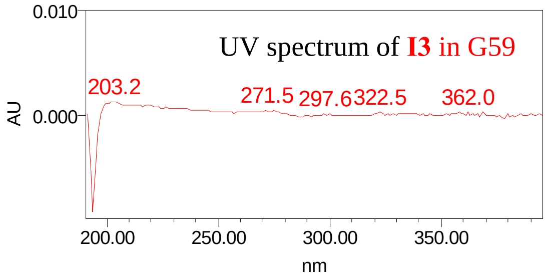

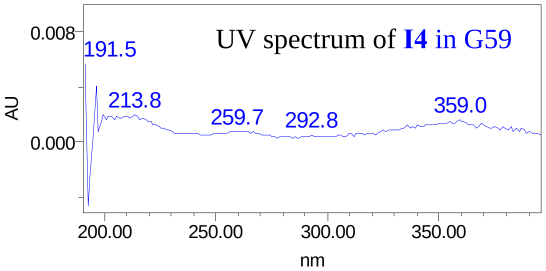

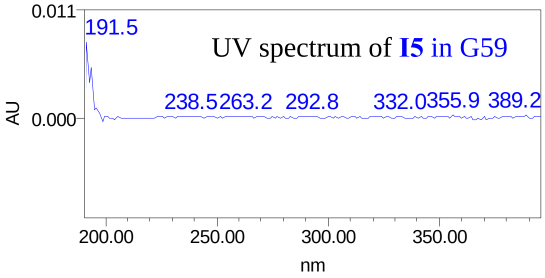

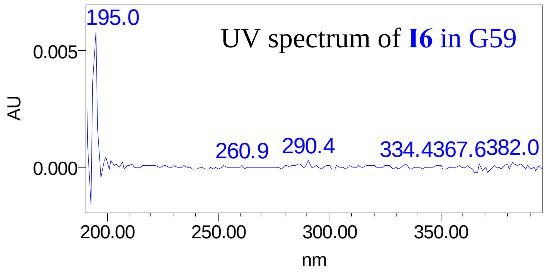

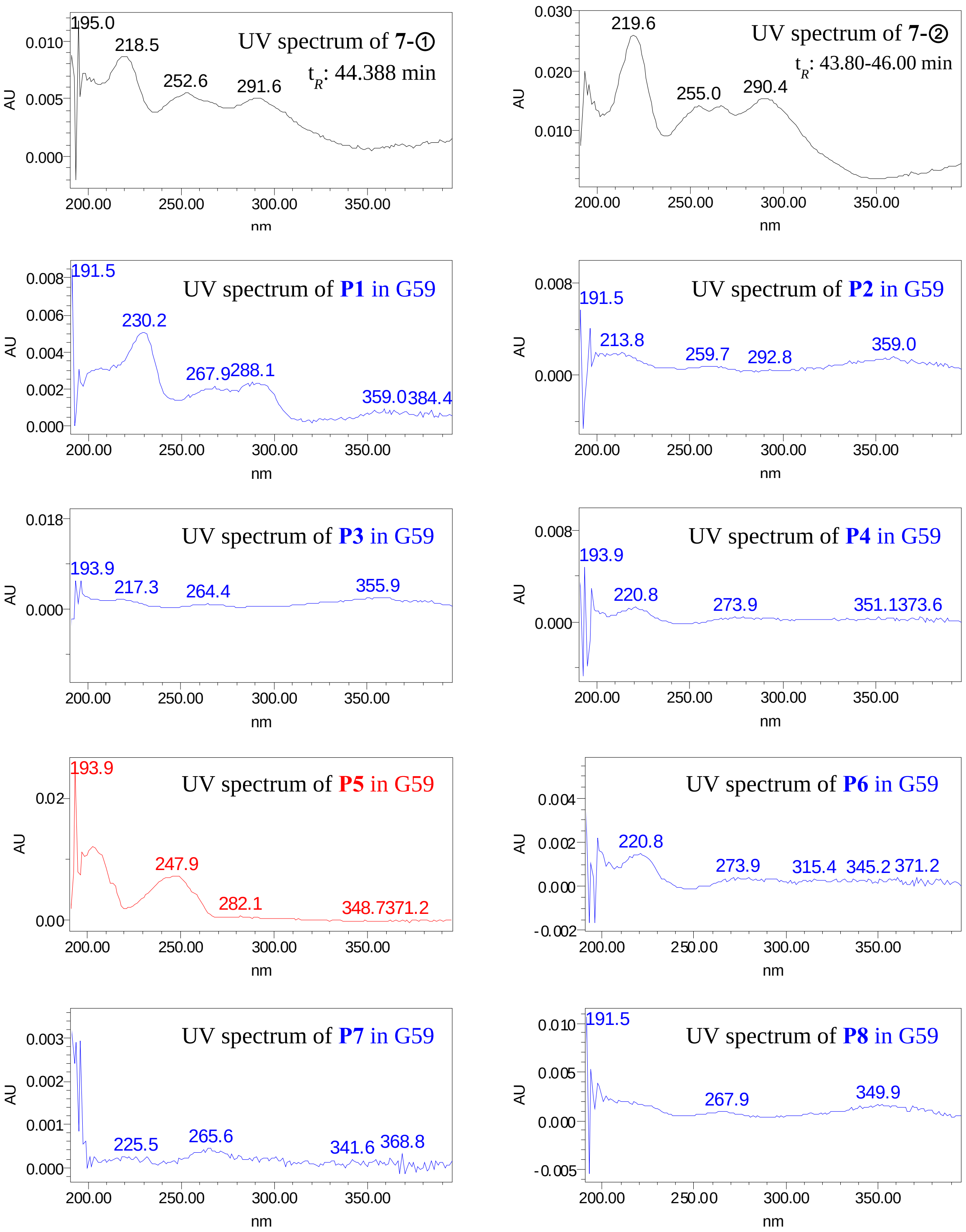
UV spectrum of 7-①
tR: 44.388 min
195.0
218.5
252.6
291.6
UV spectrum of 7-②
tR: 43.80-46.00 min
219.6
255.0
290.4
UV spectrum of P1 in G59
191.5
230.2
267.9
288.1
359.0
384.4
UV spectrum of P2 in G59
191.5
213.8
259.7
292.8
359.0
UV spectrum of P3 in G59
193.9
217.3
264.4
355.9
UV spectrum of P4 in G59
193.9
220.8
273.9
351.1
373.6
UV spectrum of P5 in G59
193.9
247.9
282.1
348.7
371.2
UV spectrum of P6 in G59
220.8
273.9
315.4
345.2
371.2
UV spectrum of P7 in G59
225.5
265.6
341.6
368.8
UV spectrum of P8 in G59
191.5
267.9
349.9
AU
nm
200.00
250.00
300.00
350.00
