# Supplementary material for: Activation of the Dormant Secondary Metabolite Production by Introducing Gentamicin-Resistance in a Marine-Derived Penicillium purpurogenum G59
Source: Mar Drugs. 2012 Mar 2;10(3):559–82. doi: 10.3390/md10030559 (PMC3347015; doi:10.3390/md10030559)

Activation of the dormant secondary metabolite production by introducing gentamicin-resistance in a marine-derived *Penicillium purpurogenum* G59

Yun-Jing Chai, Cheng-Bin Cui *, Chang-Wei Li, Chang-Jing Wu, Cong-Kui Tian and Wei Hua

Prof. Dr. C.-B. Cui, Beijing Institute of Pharmacology and Toxicology, 27 Tai-Ping Road, Haidian District, Beijing 100850, China. E-mail: cuicb@sohu.com, cuicb@126.com; Tel./Fax.: +86-10-6821- 1656.

Supplementary Data S1 – TLC and HPLC Analysis of EtOAc Extracts of G59 and Its Nine Mutant Cultures

Contents of Supplementary Data S1

1. HPLC profiles of EtOAc extracts of G59 and its nine mutant cultures…………… page S1-1
   1.1. Conditions for HPLC performance ………………… …………………… page S1-1
   1.2. HPLC profiles detected at 210 nm……………………………………… page S1-2
   1.3. HPLC profiles detected at 245 nm……………………………………… page S1-5
   1.4. HPLC profiles detected at 290 nm……………………………………… page S1-8
   1.5. HPLC profiles detected at 350 nm……………………………………… page S1-11
2. TLC Chromatograms of EtOAc extracts of G59 and its nine mutant cultures …… page S1-14

1. HPLC Profiles of EtOAc Extracts of G59 and Its Nine Mutant Cultures

1.1. Conditions for HPLC Performance

HPLC equipment: Waters HPLC system equipped with Waters 600 controller, Waters 600 pump, Waters 2414 refractive index detector, Waters 2996 photodiode array (PDA) detector and Waters Empower™ software.

HPLC Column: Venusil MP C18 column (5 µm, 100 Å, 4.6 mm × 250 mm; Agela Technologies).

Column temperature: 25°C

Mobile phase: MeOH–H_2O in linear gradient (20% MeOH at initial time 0 min → 100% MeOH at 60 min → 100% MeOH at 90 min).

Flow rate: 1 ml/min

Data processing: The Data for each HPLC chromatogram at the given wave length were extracted from the acquired PDA data and HPLC profiles were processed using Empower™ PDA software.

1.2. HPLC Profiles Detected at 210 nm

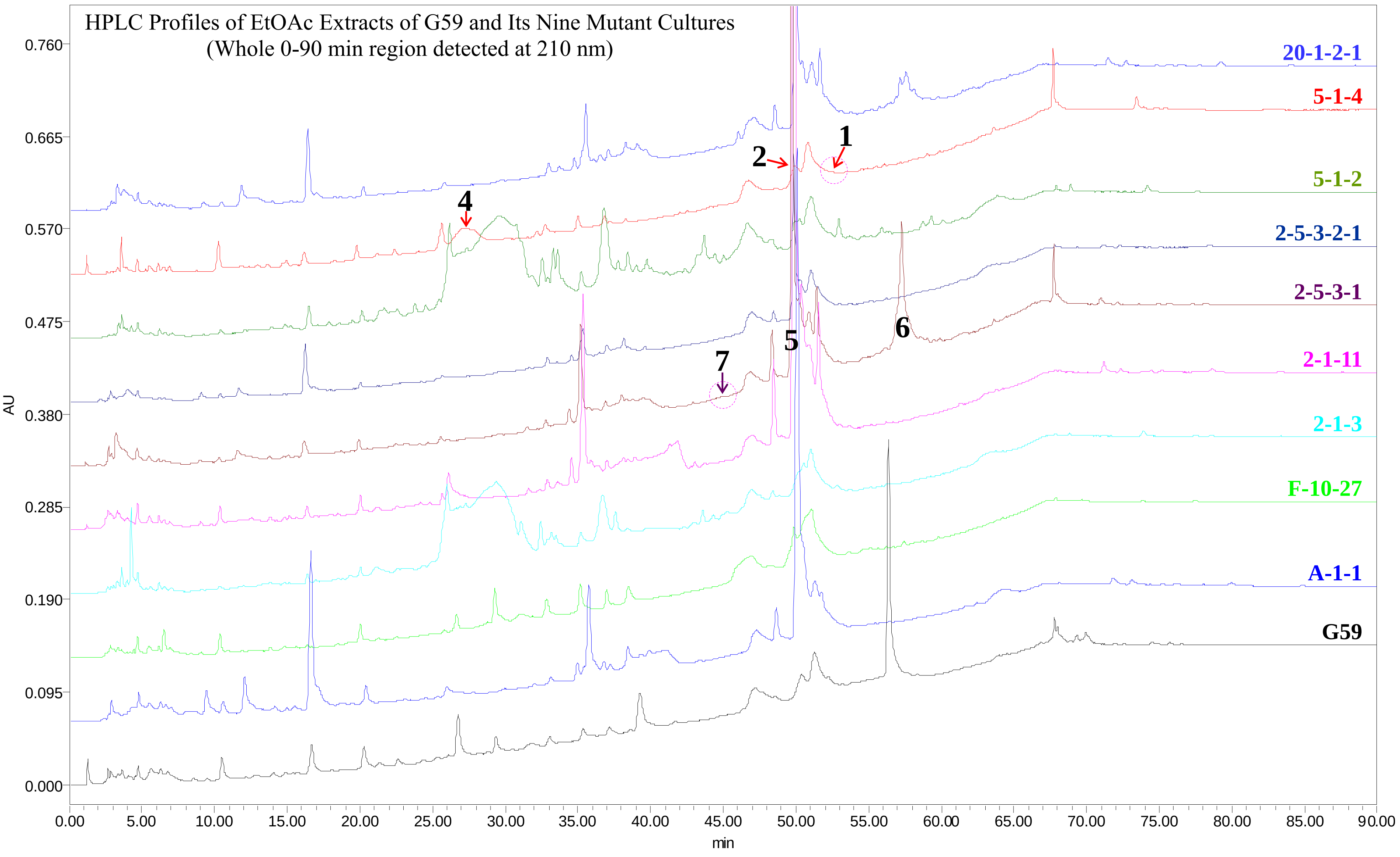

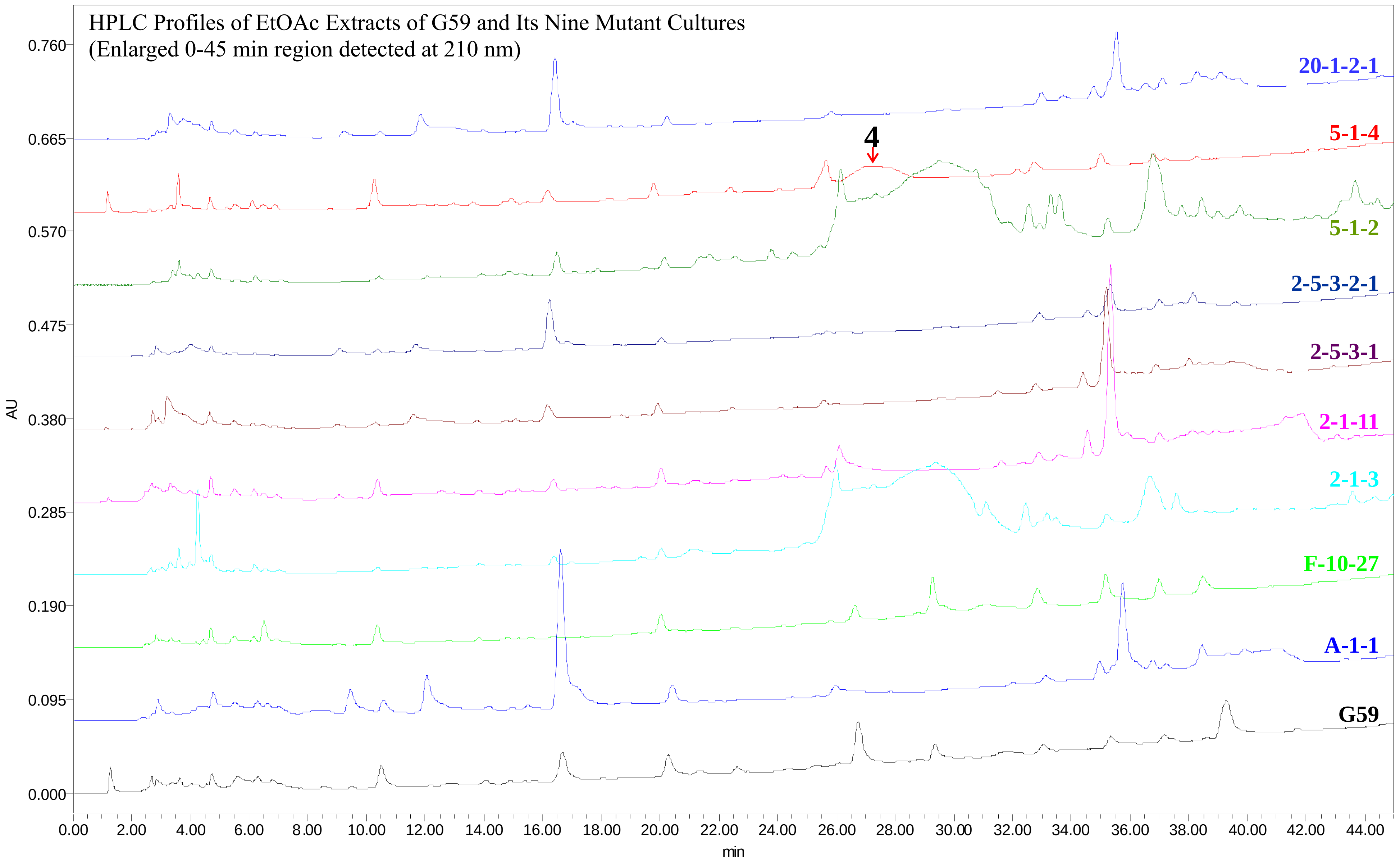
HPLC Profiles of EtOAc Extracts of G59 and Its Nine Mutant Cultures
(Enlarged 0-45 min region detected at 210 nm)
4
20-1-2-1
5-1-4
5-1-2
2-5-3-2-1
2-5-3-1
2-1-11
2-1-3
F-10-27
A-1-1
G59
AU
0.760
0.665
0.570
0.475
0.380
0.285
0.190
0.095
0.000
0.00
2.00
4.00
6.00
8.00
10.00
12.00
14.00
16.00
18.00
20.00
22.00
24.00
26.00
28.00
30.00
32.00
34.00
36.00
38.00
40.00
42.00
44.00
min

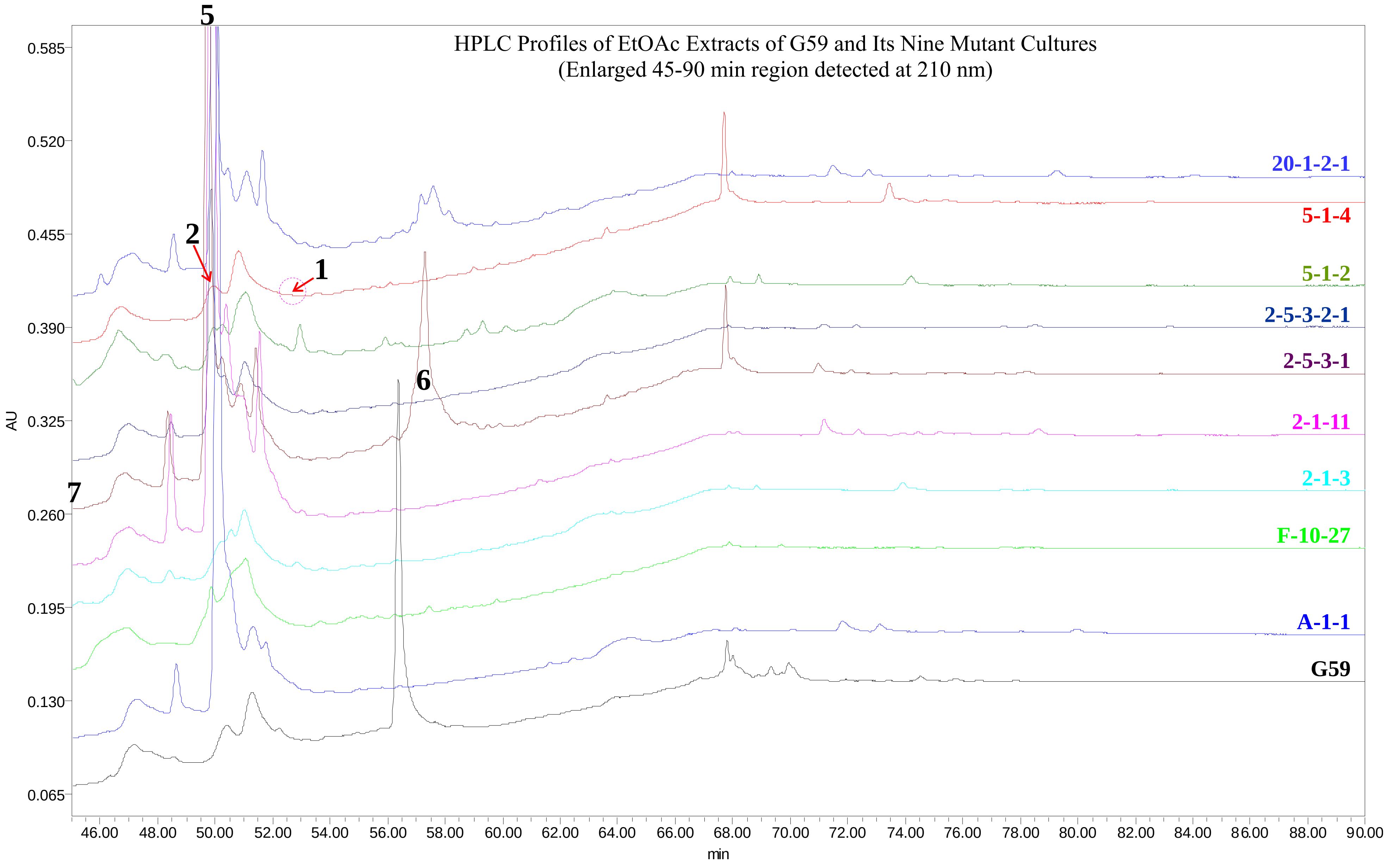
HPLC Profiles of EtOAc Extracts of G59 and Its Nine Mutant Cultures
(Enlarged 45-90 min region detected at 210 nm)
AU
0.585
0.520
0.455
0.390
0.325
0.260
0.195
0.130
0.065
46.00
48.00
50.00
52.00
54.00
56.00
58.00
60.00
62.00
64.00
66.00
68.00
70.00
72.00
74.00
76.00
78.00
80.00
82.00
84.00
86.00
88.00
90.00
min
5
2
1
6
7
20-1-2-1
5-1-4
5-1-2
2-5-3-2-1
2-5-3-1
2-1-11
2-1-3
F-10-27
A-1-1
G59

1.3. HPLC Profiles Detected at 254 nm

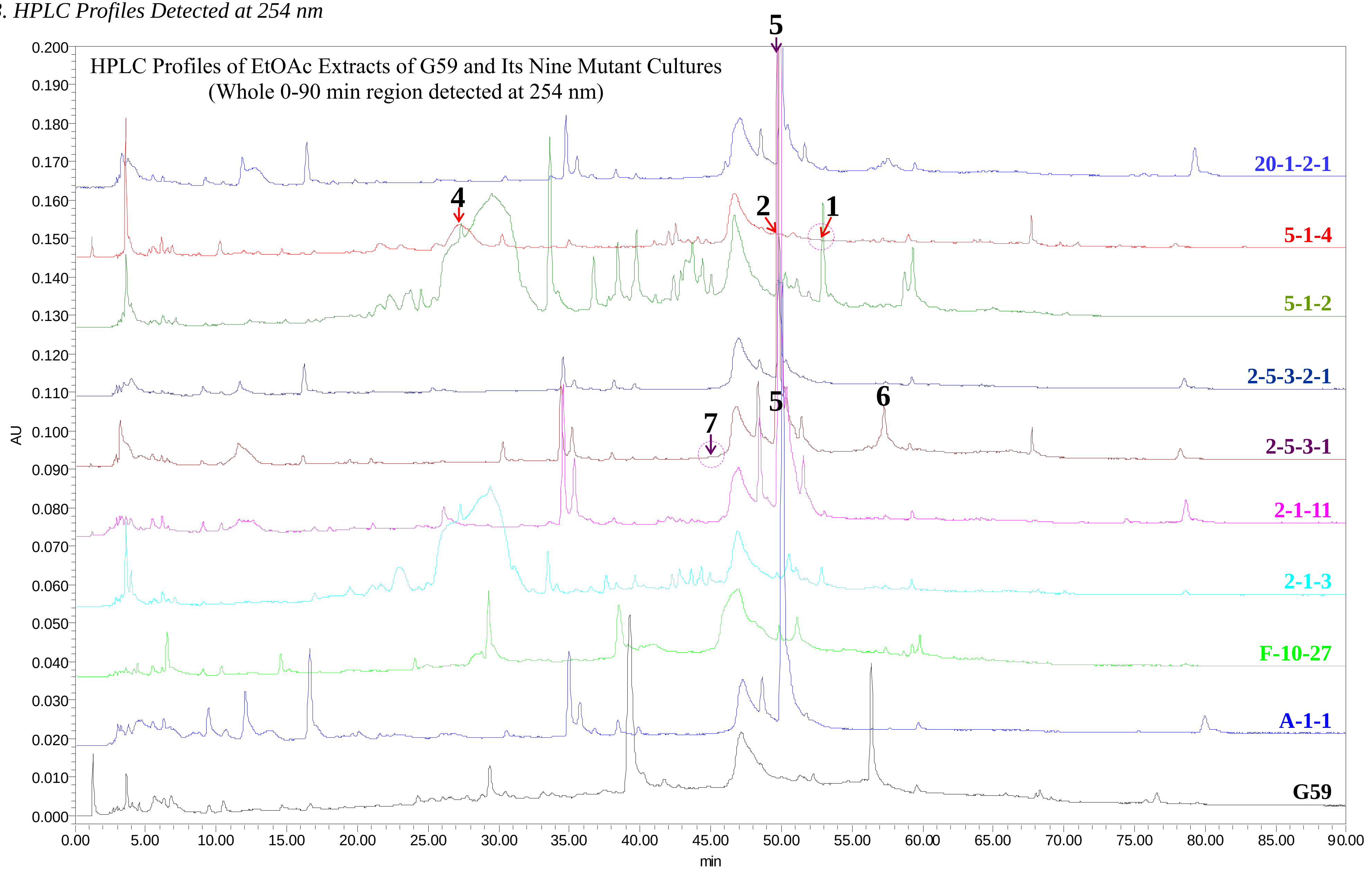

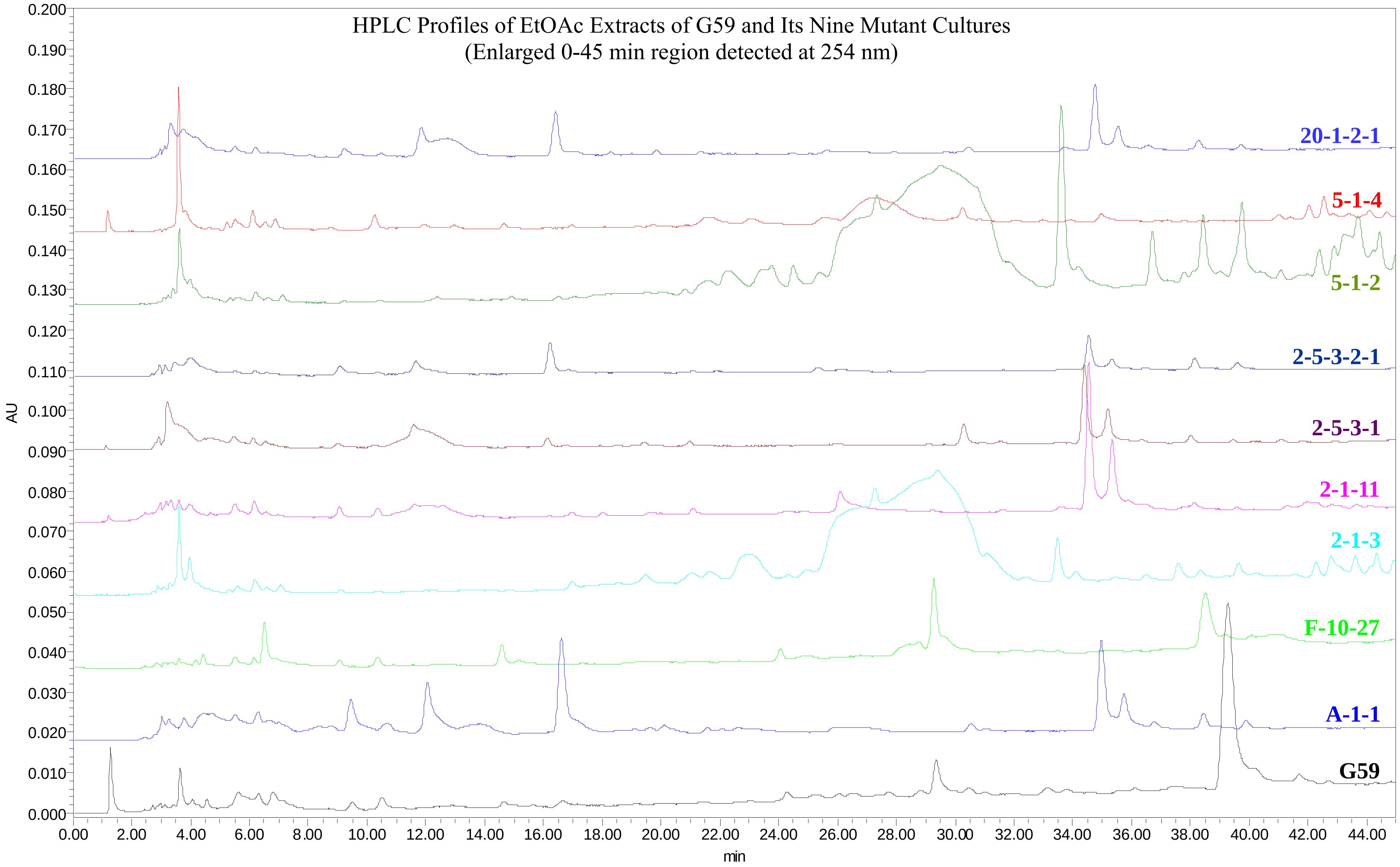
HPLC Profiles of EtOAc Extracts of G59 and Its Nine Mutant Cultures
(Enlarged 0-45 min region detected at 254 nm)
AU
min
20-1-2-1
5-1-4
5-1-2
2-5-3-2-1
2-5-3-1
2-1-11
2-1-3
F-10-27
A-1-1
G59

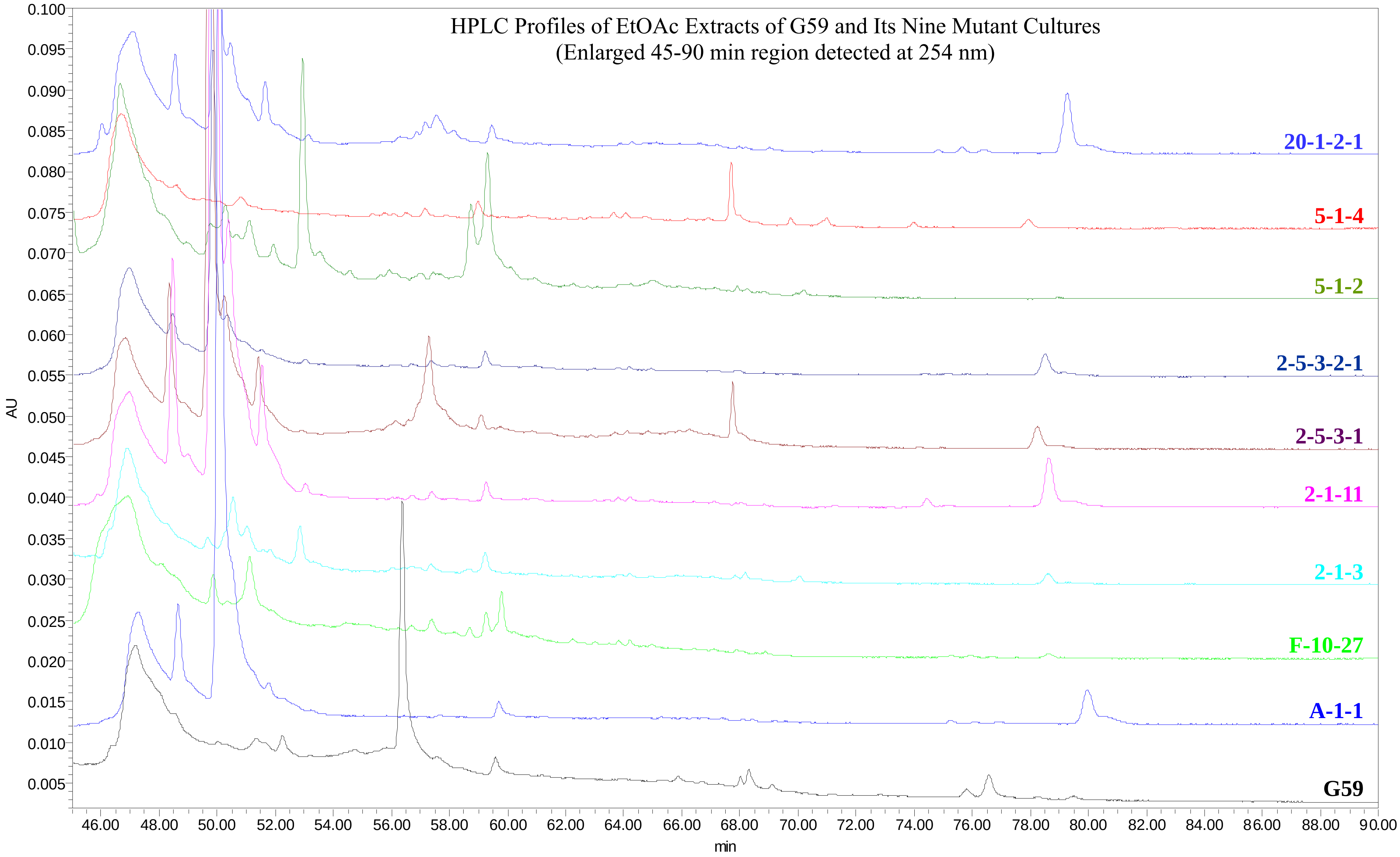
HPLC Profiles of EtOAc Extracts of G59 and Its Nine Mutant Cultures
(Enlarged 45-90 min region detected at 254 nm)
AU
min
20-1-2-1
5-1-4
5-1-2
2-5-3-2-1
2-5-3-1
2-1-11
2-1-3
F-10-27
A-1-1
G59

1.4. HPLC Profiles Detected at 290 nm

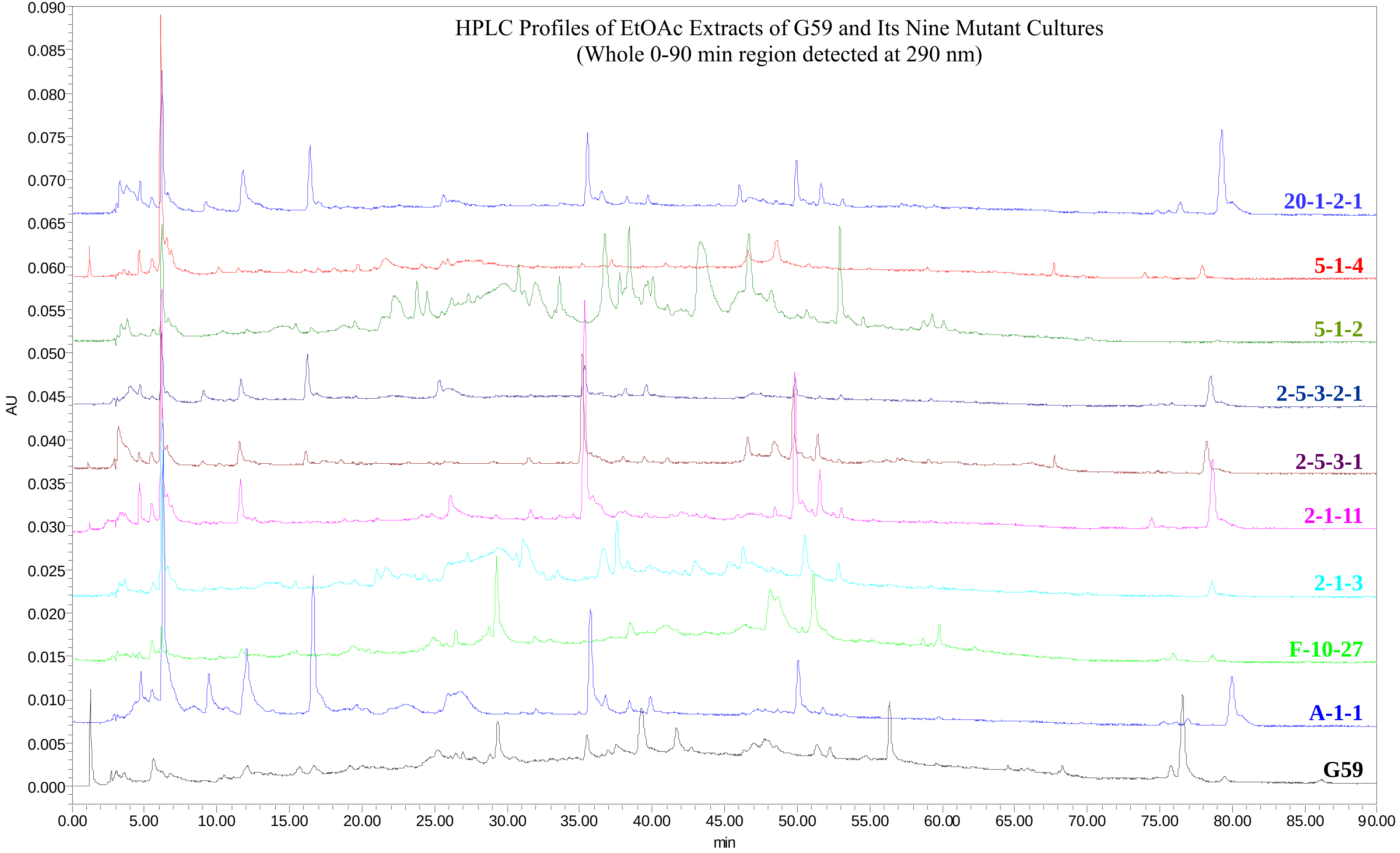

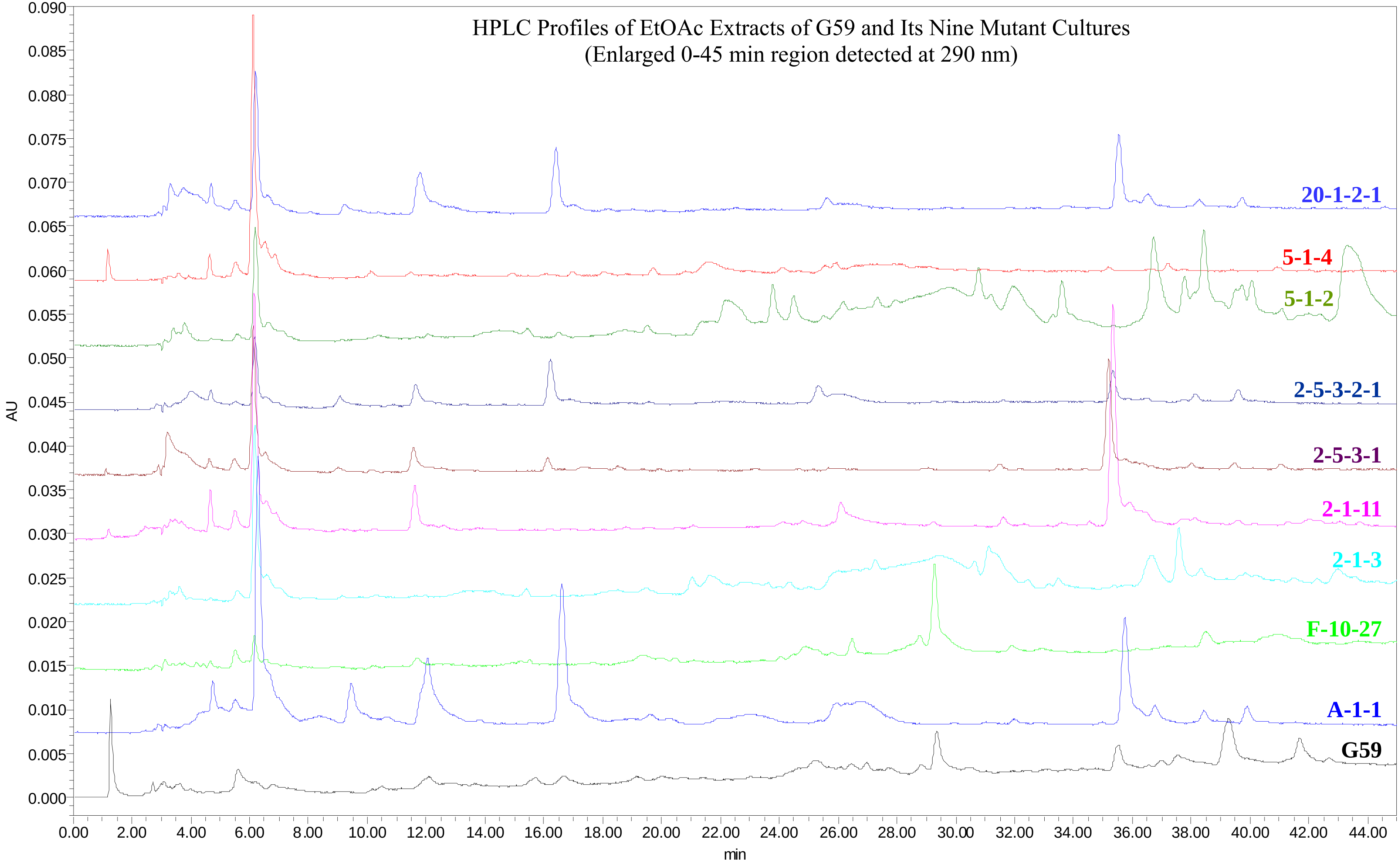

HPLC Profiles of EtOAc Extracts of G59 and Its Nine Mutant Cultures
(Enlarged 0-45 min region detected at 290 nm)
AU
min
0.090
0.085
0.080
0.075
0.070
0.065
0.060
0.055
0.050
0.045
0.040
0.035
0.030
0.025
0.020
0.015
0.010
0.005
0.000
0.00
2.00
4.00
6.00
8.00
10.00
12.00
14.00
16.00
18.00
20.00
22.00
24.00
26.00
28.00
30.00
32.00
34.00
36.00
38.00
40.00
42.00
44.00
20-1-2-1
5-1-4
5-1-2
2-5-3-2-1
2-5-3-1
2-1-11
2-1-3
F-10-27
A-1-1
G59

S1- 9 /S1-14

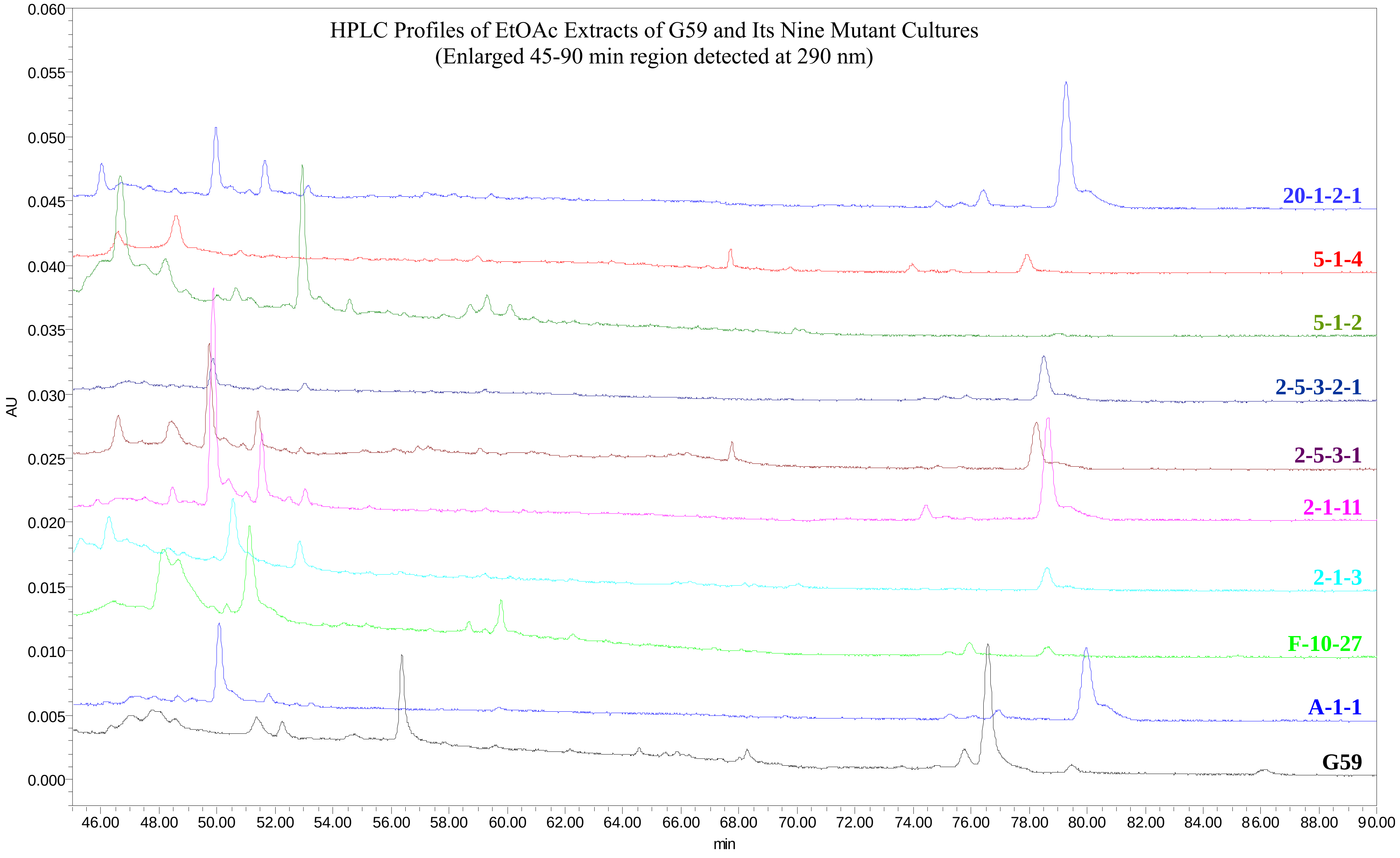

HPLC Profiles of EtOAc Extracts of G59 and Its Nine Mutant Cultures
(Enlarged 45-90 min region detected at 290 nm)
AU
min
0.060
0.055
0.050
0.045
0.040
0.035
0.030
0.025
0.020
0.015
0.010
0.005
0.000
46.00
48.00
50.00
52.00
54.00
56.00
58.00
60.00
62.00
64.00
66.00
68.00
70.00
72.00
74.00
76.00
78.00
80.00
82.00
84.00
86.00
88.00
90.00
20-1-2-1
5-1-4
5-1-2
2-5-3-2-1
2-5-3-1
2-1-11
2-1-3
F-10-27
A-1-1
G59

S1- 10 /S1-14

1.5. HPLC Profiles Detected at 350 nm

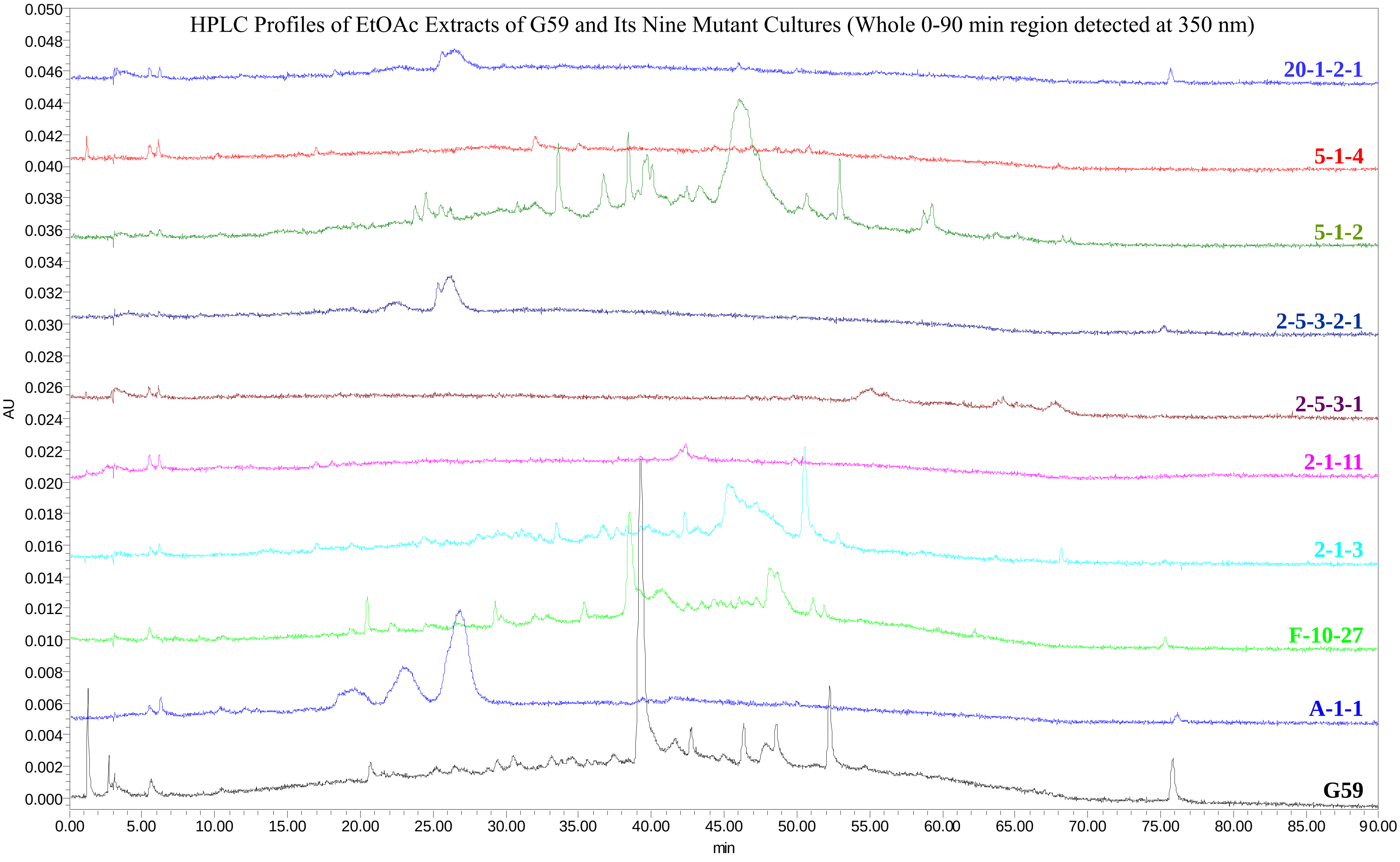

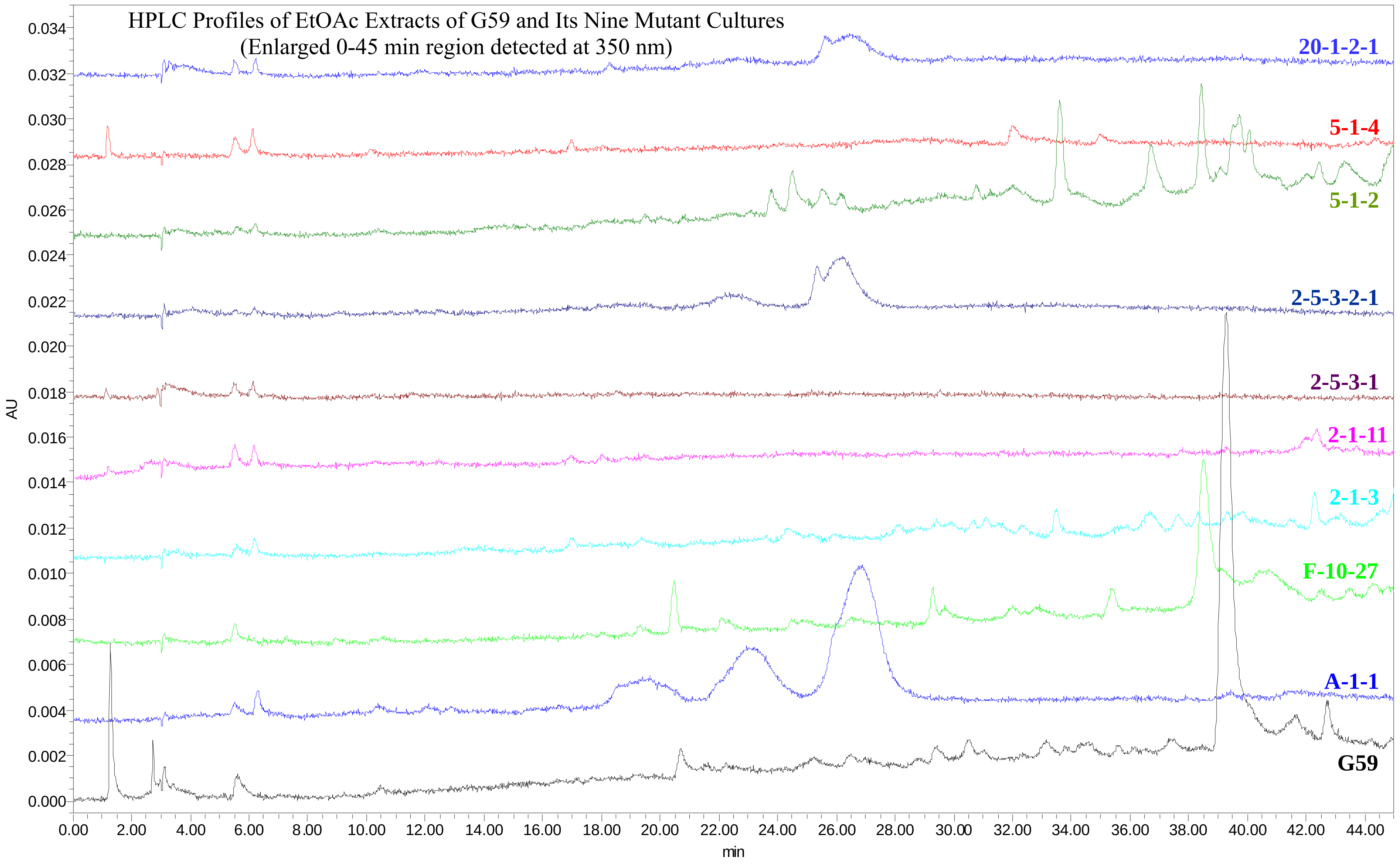
HPLC Profiles of EtOAc Extracts of G59 and Its Nine Mutant Cultures
(Enlarged 0-45 min region detected at 350 nm)
20-1-2-1
5-1-4
5-1-2
2-5-3-2-1
2-5-3-1
2-1-11
2-1-3
F-10-27
A-1-1
G59
AU
0.034
0.032
0.030
0.028
0.026
0.024
0.022
0.020
0.018
0.016
0.014
0.012
0.010
0.008
0.006
0.004
0.002
0.000
0.00
2.00
4.00
6.00
8.00
10.00
12.00
14.00
16.00
18.00
20.00
22.00
24.00
26.00
28.00
30.00
32.00
34.00
36.00
38.00
40.00
42.00
44.00
min

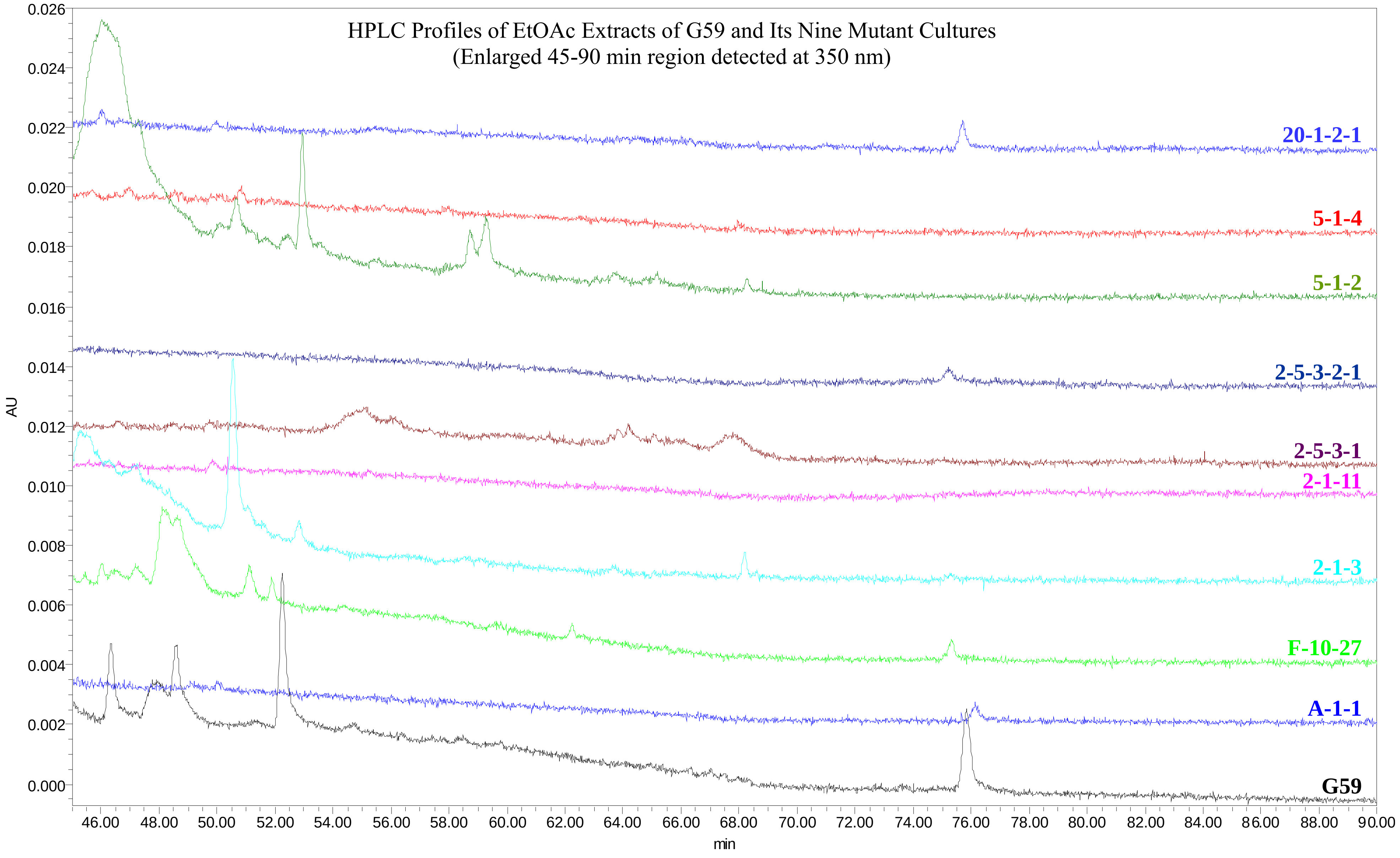
HPLC Profiles of EtOAc Extracts of G59 and Its Nine Mutant Cultures
(Enlarged 45-90 min region detected at 350 nm)
AU
min
20-1-2-1
5-1-4
5-1-2
2-5-3-2-1
2-5-3-1
2-1-11
2-1-3
F-10-27
A-1-1
G59
0.026
0.024
0.022
0.020
0.018
0.016
0.014
0.012
0.010
0.008
0.006
0.004
0.002
0.000
46.00
48.00
50.00
52.00
54.00
56.00
58.00
60.00
62.00
64.00
66.00
68.00
70.00
72.00
74.00
76.00
78.00
80.00
82.00
84.00
86.00
88.00
90.00

2. TLC Chromatograms of EtOAc Extracts of G59 and Its Nine Mutant Cultures

Plate: Silica gel GF_{254}. Developing solvent: $CHCl_3$–MeOH (9:1). Detection: left, UV 254 nm; middle, UV 365 nm; right, Vaughan's reagent.

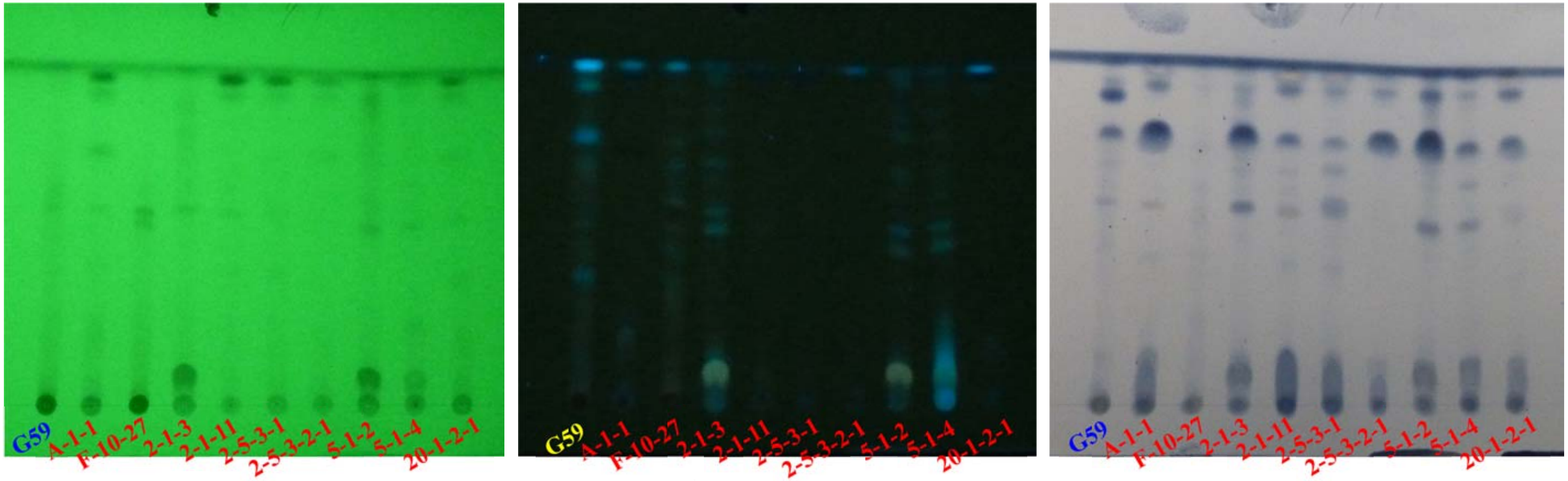

Plate: Silica gel GF_{254}. Developing solvent: $CHCl_3$–MeOH (15:1). Detection: left, UV 254 nm; middle, UV 365 nm; right, Vaughan's reagent.

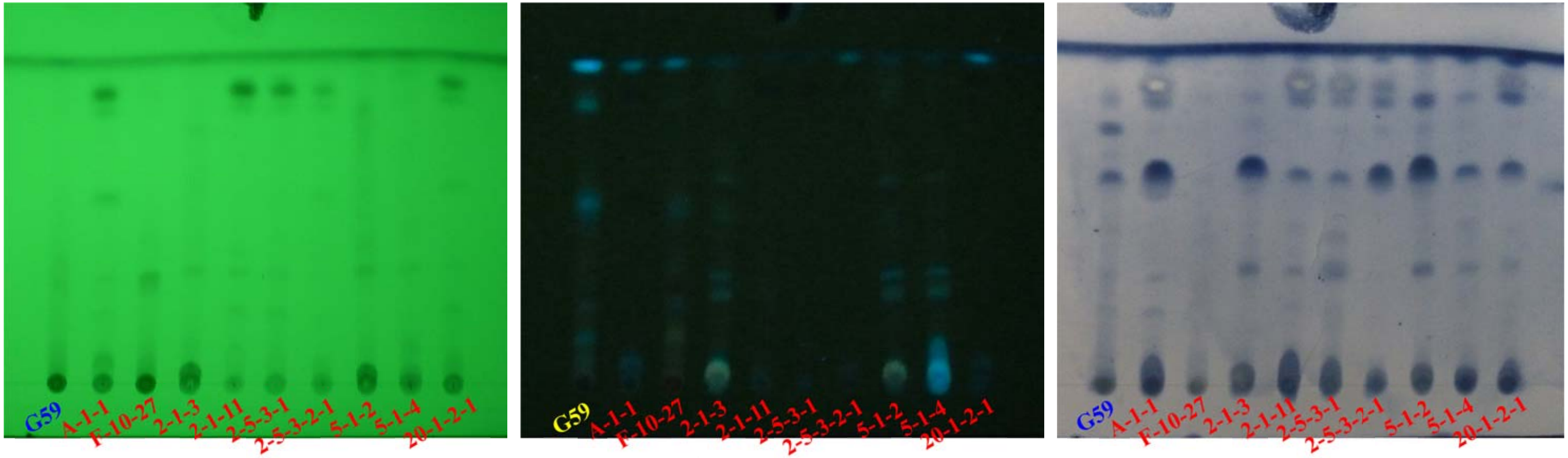

Supplement: Supplementary File 1: — ZIP-Document (ZIP, 1158 KB) [file marinedrugs-10-00559-s001.zip › marinedrugs-13100-supplementary/Supplementary Data S1 - TLC and HPLC analysis of EtOAc Extracts from G59 and Nine Mutants.pdf]
